# The healing performance of aerobic-anaerobic composite microbial self-healing recycled concrete under oxygen enrichment-oxygen deficient environment

**DOI:** 10.1128/aem.00938-26

**Published:** 2026-06-15

**Authors:** Yonggui Wang, Pengfei Li, Xin Fang, Xingguo Wang, Doh Shu Ing

**Affiliations:** 1 School of Civil Engineering, Henan Polytechnic University 12561, Jiaozuo, China; 2 Henan Province Engineering Laboratory for Eco-architecture and the Built Environment, Jiaozuo, China; 3 Faculty of Civil Engineering Technology, University Malaysia Pahang Al-Sultan Abdullah, Pahang, Malaysia; Colorado School of Mines, Golden, Colorado, USA

**Keywords:** composite microorganisms, recycled concrete, mechanical properties, microstructure

## Abstract

Concrete inevitably develops cracks during service, which seriously impairs the functional performance of structures. Microbially induced calcium carbonate precipitation technology has gained attention for its eco-friendliness, cost-effectiveness, and potential for crack healing. This study used carbonated recycled fine aggregates to immobilize Bacillus cohnii and Nitratireductor indicus for preparing a microbial repair agent. Microbial self-healing recycled concrete was made by controlling the repair agent dosage and recycled coarse aggregate replacement ratio. Macroscopic performance tests were conducted to analyze the compressive, splitting, and flexural strengths of self-healing recycled concrete and assess crack healing effectiveness at various mineralization times. Combined with microscopic tests, the micro-morphology and crystal types of mineralized precipitates under different oxygen contents were analyzed. The findings show that increasing the microbial repair agent dosage reduces the concrete’s strength, while significantly improving the crack healing ratio. After 28 days of healing, when the repair agent dosage was 4%, the crack healing ratio for 0.3 mm width cracks in the oxygen-rich zone, transition zone, and oxygen-deficient zone was 100.00%, 93.30%, and 96.70%, respectively. However, for 0.7 mm width cracks, the highest healing ratio of only 38.57% occurred in the oxygen-rich zone. The healing effectiveness decreases with increasing crack width. Regarding the influence of oxygen content, the healing performance is optimal in the oxygen-rich zone, followed by the oxygen-deficient zone, and least effective in the transition zone. Under different oxygen environments, the mineralized precipitates were all calcite-type CaCO_3_. This study offers theoretical support for prolonging the service life of recycled concrete.

**IMPORTANCE:**

Based on the fact that in concrete projects under humid environments, the surface of slender cracks in concrete engineering is relatively high, while the oxygen content in the deep cracks is low, this study immobilizes a mixture of aerobic and anaerobic microorganisms in recycled fine aggregates. By analyzing the crack repair performance of microbially self-healing recycled concrete under conditions where oxygen-rich and oxygen-poor areas coexist, as well as the mineralization mechanism of mixed microorganisms, this research can provide a theoretical basis and technical support for the repair performance of recycled concrete under external loads. The research conclusions are significant for the engineering application of microorganisms and the resource utilization of construction waste. At the same time, it has important practical significance for extending the service life of concrete engineering.

## INTRODUCTION

Concrete, as a multiphase heterogeneous material widely used in the field of construction engineering, has the disadvantages of low tensile strength and high brittleness (1, 2), and its performance deteriorates over time and with environmental corrosion (3). During the service stage, microcracks or localized damage are prone to form within the interior or on the surface of concrete due to autogenous shrinkage, temperature fluctuations, and external loading (2). The formation of cracks provides pathways for aggressive agents to penetrate the concrete, leading to steel reinforcement corrosion and progressive degradation of the concrete structure. Therefore, the generation of microcracks is a primary cause of reduced concrete durability (4), especially in critical infrastructure, such as dams, marine structures, and tunnels, that operate long-term in harsh environments, where microcrack propagation severely threatens structural safety and service life (5).

Traditional concrete repair methods are passive post-repair approaches, such as surface coating (6) and pressure grouting (7). These methods are not only complicated and costly to implement, but are mainly effective for wide cracks in concrete, making it difficult to repair microcracks (8). Microbial self-healing not only has self-diagnosis and self-healing capabilities but also effectively addresses the compatibility issue between crack fillers and the concrete matrix (9–12). However, the high alkalinity inside concrete and cement hydration adversely affects microorganisms (13), necessitating the use of carriers for microbial immobilization (14, 15). Existing research has shown that porous recycled brick aggregates as carriers successfully achieved crack healing in concrete (16). Domestic scholars have also systematically reviewed microbial immobilization-encapsulation technology, highlighting its promising application prospects (17). At present, a range of carrier materials, such as microcapsules (18), silica gel (19), hydrogel (20), polyurethane (19), diatomite (21–23), and expanded perlite (8), have been widely used for bacterial immobilization. These approaches can effectively enhance microbial mineralization activity and crack healing performance (24).

There are various types of microorganisms suitable for use in self-healing concrete. Based on past research, there are mainly four types of microbial self-healing systems (1). The first type is a self-healing system based on urease-producing bacteria. These bacteria decompose urea through their own urease to induce the precipitation of calcium carbonate, thereby repairing concrete cracks (25). The second is the aerobic bacterial system. Aerobic bacteria can convert the nutrients pre-added during the concrete preparation process (such as calcium lactate) into CaCO_3_ precipitates through their own metabolism (26). The third is the denitrifying bacteria system, where denitrifying bacteria reduce nitrate (NO_3_^−^) to nitrogen gas, generating CO_3_^2−^ and HCO_3_^−^ ions, which then react with free Ca^2+^ to form CaCO_3_ precipitates (4). The fourth is the carbonic anhydrase bacteria system, where such bacteria can capture CO_2_ from the air via self-produced carbonic anhydrase and convert it to HCO_3_^−^, thereby promoting CaCO_3_ precipitation (27, 28).

Based on this, this study crushed foam concrete to produce recycled fine aggregates, carbonized them, adsorbed the aerobic Bacillus cohnii and anaerobic Nitratireductor indicus onto the carbonized recycled fine aggregates, and then coated them with a protective layer to prepare repair agents. Microbial self-healing recycled concrete (hereinafter referred to as self-healing recycled concrete) was prepared with the repair agent dosage and recycled coarse aggregate replacement ratio as mix proportion control parameters. Through macroscopic loading tests, the structural performance of self-healing recycled concrete was evaluated at various repair agent dosages and replacement ratios. Combined with artificially created cracks, the healing effectiveness of self-healing recycled concrete under different repair agent dosages was analyzed. Using micro-scale characterization techniques, such as Scanning Electron Microscopy (SEM), Energy-Dispersive Spectroscopy (EDS), X-ray Diffraction (XRD), Atomic Force Microscopy (AFM), and Thermogravimetric Analysis–Differential Scanning Calorimetry (TG-DSC), the micro-morphology, elemental composition, and crystal types of the mineralized precipitates in self-healing recycled concrete were analyzed. Meanwhile, the ultrasonic pulse velocity (UPV) test, as a non-destructive testing method, was used to evaluate the internal compactness and healing effectiveness of the self-healing recycled concrete. The findings of this research offer valuable references for improving the durability of buildings and extending their service life.

## MATERIALS AND METHODS

In this study, the term “curing” is used to emphasize the time-dependent process (e.g., curing age), whereas “healing” is used to describe the crack repair process and its performance (e.g., healing ratio).

### Material properties

**Fig 1:**
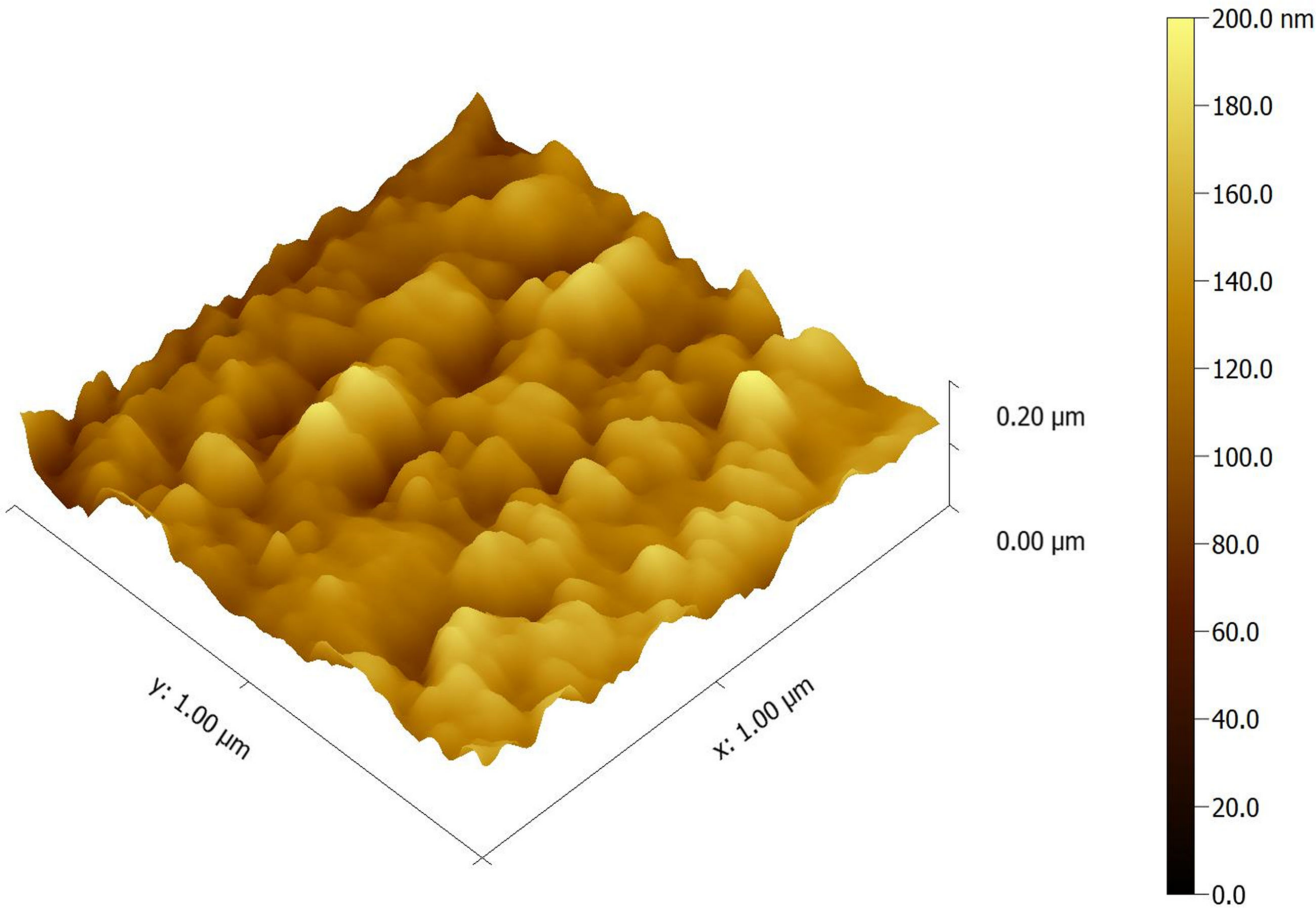
AFM image after BF modification.

The cement selected was Ordinary Portland Cement (P.O 42.5) produced by Jiaozuo Qianye Cement Co., Ltd., and its performance indicators complied with the requirements of “Ordinary Portland Cement” (GB 178-2007). Basalt fibers (BF) were purchased from Haining Anjie Composite Materials Co., Ltd. Due to their ability to restrain microcrack propagation through a crack-bridging effect and enhance the mechanical properties of concrete, BF was incorporated as a reinforcing material. The AFM image of basalt fibers after treatment with 2.91% KH550 and 5% NS modification solution is shown in Fig. 1. It was evident that after treatment with the modification solution, the fiber surface exhibited a hilly undulating morphology, indicating a good modification effect. Nano silica was purchased from Hebei Enyi Metal Powder Research Institute, with a particle size of 10 nm, and was used for the modification treatment of BF. The coarse aggregates included natural coarse aggregate (NA) and recycled coarse aggregate (RA). NA consisted of continuously graded crushed stone, and its physical properties are presented in Table 1. RA was obtained by crushing and screening *in situ* cast and 28-day cured C40 concrete components, with its performance parameters as shown in Table 1. The fine aggregates included natural fine aggregate (NFA) and recycled fine aggregate (RFA). NFA was medium-grained river sand with a fineness modulus of 2.73. RFA was obtained by crushing and sieving foam concrete cast in the Structural Test Hall of Henan Polytechnic University after 28 days of curing. The test water used was tap water from Jiaozuo city.

**TABLE 1:** Performance parameters of aggregate

Aggregate type	Bulk density (kg·m^−3^)	Apparent density (kg·m^−3^)	Mud content (%)	Water absorption (%)	Crushing value (%)
NA	1,477	2,751	0.54	1.47	7.6
RA	1,342	2,586	3.51	4.52	16.1
RFA	457	904	/^*a*^	24	54
CRFA	514	1,146	/	22	50

^*a*^ “/” indicates that the mud content test was not applicable.

### Mix proportion design

To investigate the healing performance of self-healing recycled concrete, the repair agent dosage and replacement ratio of recycled coarse aggregate were established as design variables for the mix proportion. The self-healing recycled concrete mix proportions are shown in Table 2. The repair agent dosage was set at three levels (3%, 4%, and 5%). To ensure uniform dispersion of modified basalt fibers and repair agent particles in the concrete matrix, a staged feeding method was adopted during concrete mixing based on preliminary research. First, cement, sand, water, and aggregates were mixed according to the Chinese standard “Standard for test method of performance on ordinary fresh concrete” (GB/T 50080-2016). The mixed concrete was then poured into plastic molds, demolded after 1 day, and the concrete specimens were placed in a standard curing chamber (temperature 20°C ± 2°C, humidity 95%) for 28 days of curing.

**TABLE 2:** Mix proportion of self-healing recycled concrete

Group	Specimen	Water (kg/m^3^)	Cement (kg/m^3^)	Sand (kg/m^3^)	BF (kg/m^3^)	NA (kg/m^3^)	RA (g/m^3^)	Repair agent dosage
1	NC	200	490	525	0	1,225	0	0
2	NC-BF	200	490	525	3	1,225	0	0
3	NC-M3	200	490	525	3	1,225	0	3%
4	NC-M4	200	490	525	3	1,225	0	4%
5	NC-M5	200	490	525	3	1,225	0	5%
6	R50-M3	200	490	525	3	612.5	612.5	3%
7	R50-M4	200	490	525	3	612.5	612.5	4%
8	R50-M5	200	490	525	3	612.5	612.5	5%
9	R100-M3	200	490	525	3	0	1,225	3%
10	R100-M4	200	490	525	3	0	1,225	4%
11	R100-M5	200	490	525	3	0	1,225	5%

### Microbial cultivation

#### Aerobic microbial cultivation

**Fig 2:**
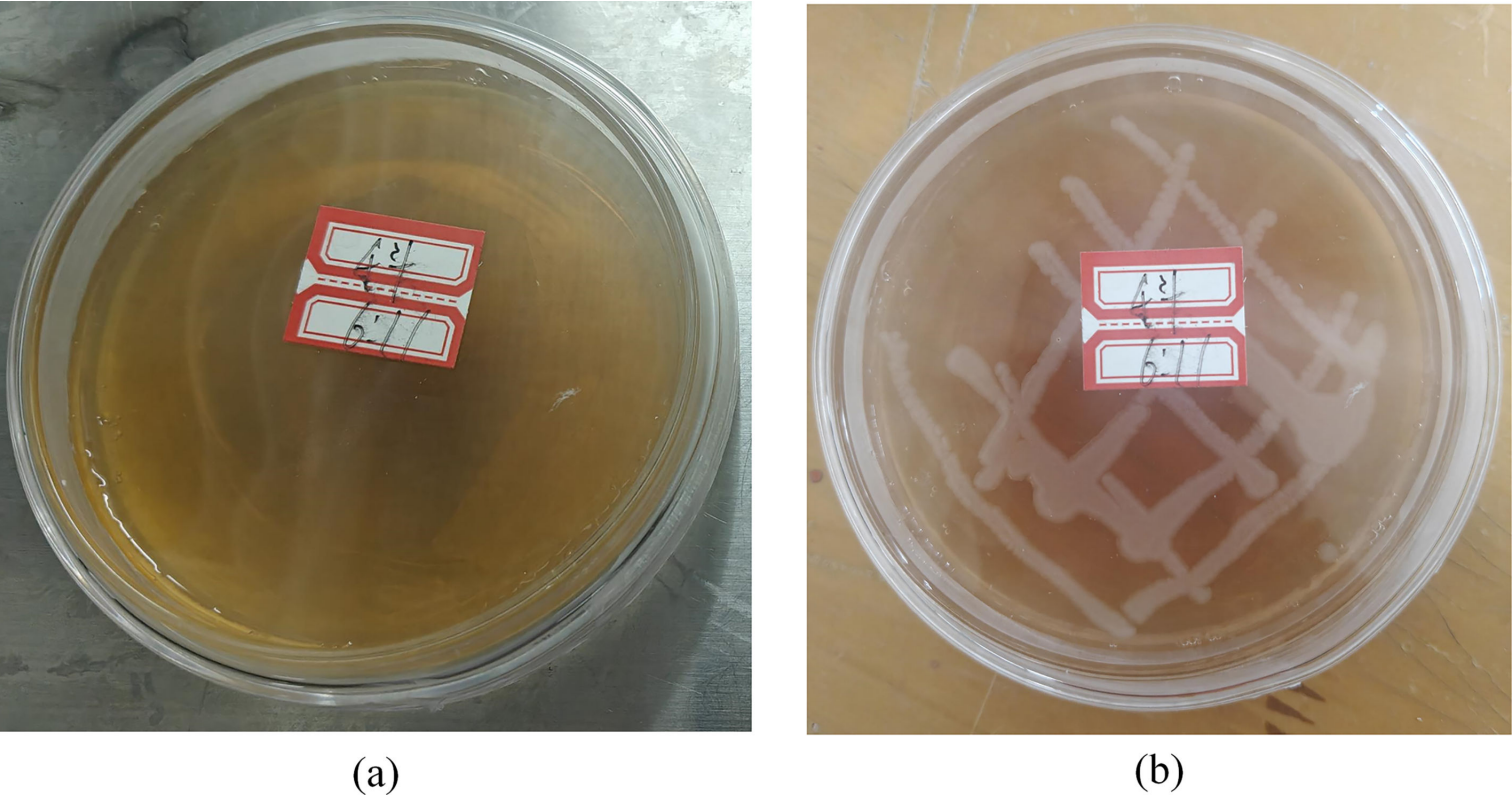
Aerobic microbial culture medium before and after cultivation: (**a**) before cultivation; (**b**) after cultivation

The aerobic microorganism used in this experiment was Bacillus cohnii (HZB197250). The freeze-dried powder of this bacterium was stored in an ampule, and its medium composition is shown in Table 3. The freeze-dried bacteria were rehydrated to prepare a bacterial suspension. The liquid medium and solid plates were sterilized under high pressure at 121°C and 0.1 MPa for 20 min, then cooled to room temperature for later use. Under sterile conditions, the bacterial suspension was inoculated onto the surface of the solid medium (Fig. 2a) and incubated at 30°C for 48 h until visible colonies formed (Fig. 2b). Subsequently, the colonies were inoculated into liquid medium for scale-up cultivation, and the culture was incubated until the bacterial suspension became turbid, indicating successful scale-up. Glycerol stocks were prepared and stored at 4°C for future use.

**TABLE 3:** Components of aerobic bacteria medium (g/L)

Tryptone	Phytone	NaCl	K_2_HPO_4_	Glucose	Agar powder
17	3	5	2.5	2.5	15

**Fig 3:**
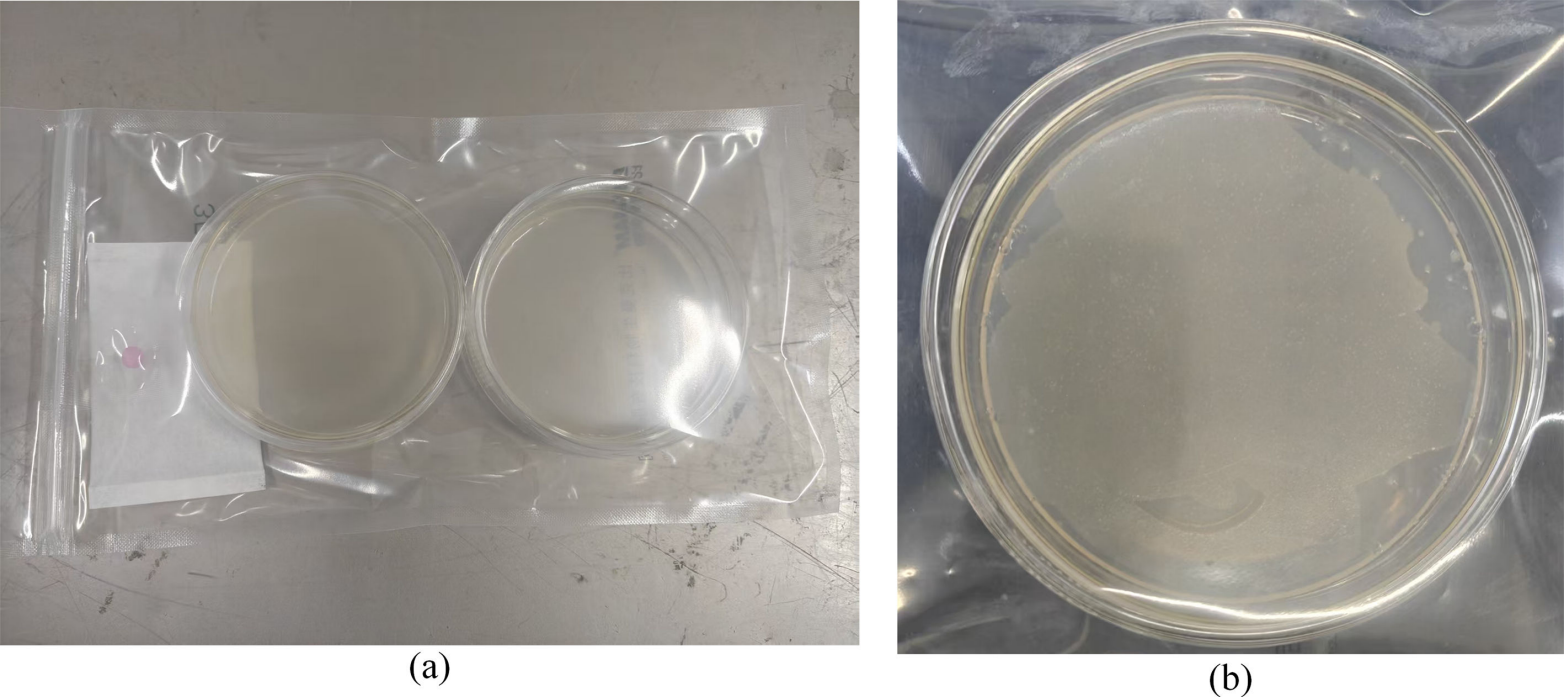
Anaerobic microbial culture medium before and after cultivation: (**a**) before cultivation; (**b**) after cultivation

#### Anaerobic microbial cultivation

The anaerobic microorganism used in this experiment was Nitratireductor indicus (HZB194461), and the medium composition is shown in Table 4. The initial cultivation steps were similar to those used for Bacillus cohnii. The inoculated solid medium was then placed in an anaerobic bag (Fig. 3a) and incubated at 28°C for 72 h until colonies formed (Fig. 3b). Subsequently, the colonies were inoculated into liquid medium for scale-up cultivation. Prior to incubation, dissolved oxygen in the liquid medium was removed by nitrogen purging for 15 min, and the culture vessels were sealed with sealing film and aluminum foil. The culture was incubated until turbidity was observed. The resulting bacterial culture was prepared as glycerol stocks and stored at 4°C for subsequent use.

**TABLE 4:** Components of anaerobic bacteria medium

Substance	Content (g)	Substance	Content (g)	Substance	Content (g)	Substance	Content (g)
Peptone	5.0	Yeast extract	1.0	Ferric citrate	0.1	NaCl	19.45
MgCl_2_	5.98	Na_2_SO_4_	3.24	CaCl_2_	1.8	KBr	0.08
KCl	0.55	Na_2_CO_3_	0.16	NH_4_NO_3_	0.0016	Na_2_HPO_4_	0.008
SrCl	0.034	Boric acid	0.022	Na_2_SiO_3_	0.004	NaF	0.0024
Agar powder	15						

#### Aerobic and anaerobic mixed cultivation

Colonies of Bacillus cohnii were inoculated into 50 mL of specialized liquid medium, and colonies of Nitratireductor indicus were similarly inoculated into another 50 mL of specialized liquid medium. The two bacterial suspensions were mixed in a conical flask to prepare a mixed bacterial suspension, which was then divided into two conical flasks for aerobic and anaerobic cultivation.

Aerobic group: the flask was sealed with breathable sealing film and placed in a 30°C constant temperature incubator for shake cultivation.

Anaerobic group: the flask was first purged with nitrogen gas for 15 min, then sealed with sealing film and aluminum foil, and placed in a 28°C constant temperature incubator for static cultivation.

The start time of cultivation was recorded as 0 h, and OD_600_ measurements were conducted every 3 h over a total period of 48 h (0–48 h). Under aseptic conditions, 1 mL of bacterial suspension was aspirated from each conical flask, and the absorbance of each sample at 600 nm was recorded with a spectrophotometer to determine the OD value.

### Experimental program

#### Preparation of composite microbial repair agent

**Fig 4:**
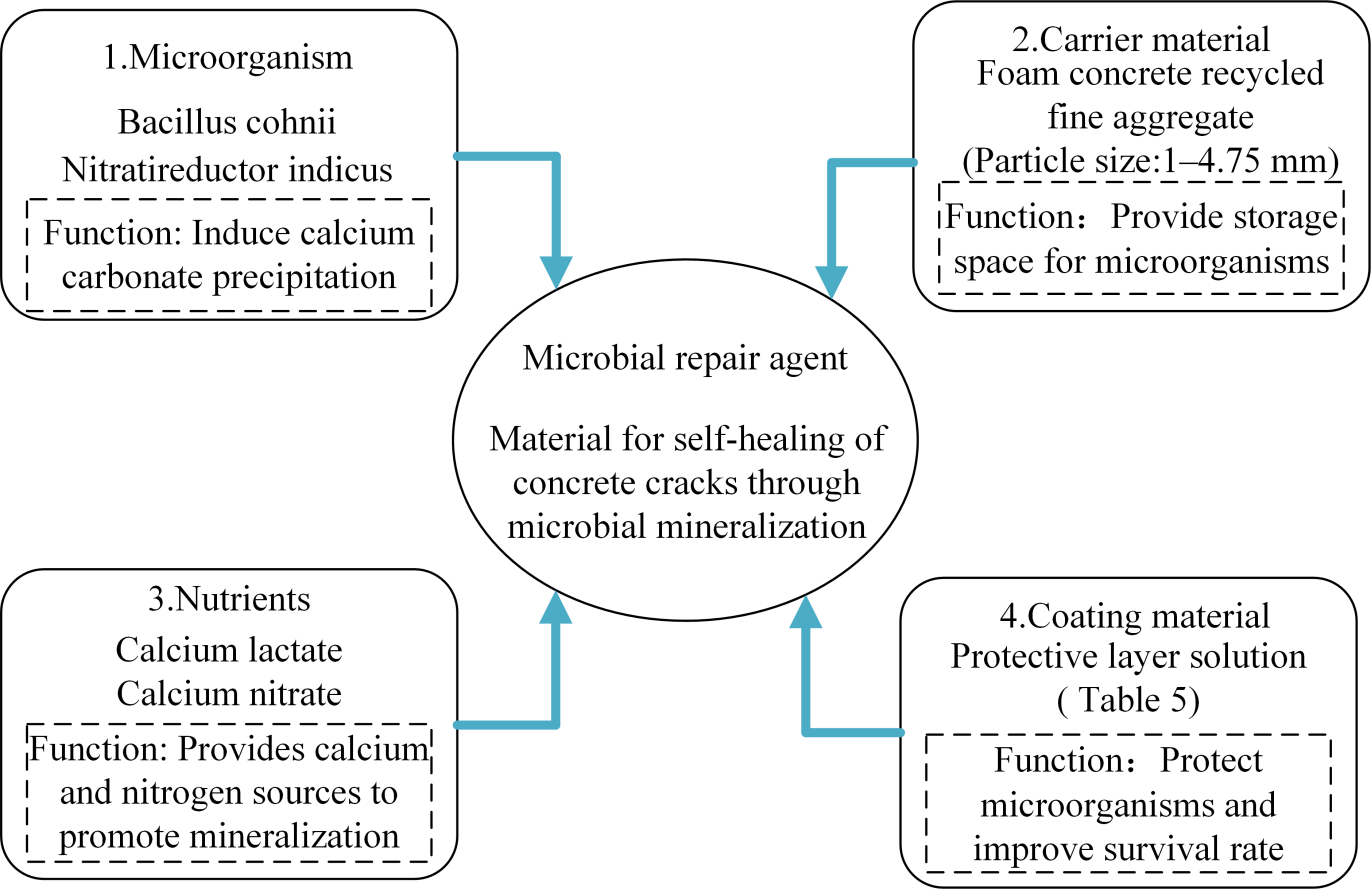
Composition and functions of the microbial repair agent.

The preparation process for the microbial repair agent mainly included the following steps:

1. Suspensions of Bacillus cohnii and Nitratireductor indicus were prepared according to the above bacterial cultivation steps.
2. The carbonated foam concrete recycled fine aggregates, along with nutrients (calcium lactate and calcium nitrate) required for growth and mineralization, were placed into a negative pressure saturation device. The vacuum pump was started to exhaust air from the device until the pressure reached −0.06 MPa. Then, the negative pressure was used to draw the prepared bacterial solution into the device. The bacterial solution and nutrients were allowed to fully soak and adsorb onto the aggregates, obtaining bacteria-loaded recycled fine aggregates.
3. The foam concrete recycled fine aggregates prepared through the above procedures were oven-dried at a constant temperature of 40°C until their mass remained unchanged.
4. The protective layer solution was prepared according to Table 5, and the dried recycled fine aggregates were coated with it. After coating, the aggregates were cured for 1 day and finally placed in a 40°C constant temperature incubator. They were then dried to obtain the microbial repair agent. The composition and functions of the microbial repair agent are shown in Fig. 4.

**TABLE 5:** Protective layer mix ratio

Alkali activator solution			Metakaolin (g)	Sodium methyl silicate (g)	Styrene-acrylic emulsion (g)	Bacteria-loaded RFA (g)	Coating effect
Sodium silicate (g)	NaOH (g)	Water (g)					
90	15	43	100	5	6	540	Particle dispersion

#### Mechanical performance test

The mechanical performance tests of this experiment mainly include compressive, splitting, and flexural strength tests. Compressive and splitting strength tests were conducted using 100 × 100 × 100 mm cubic specimens, while flexural strength tests were performed on 100 × 100 × 400 mm specimens. The compressive, splitting, and flexural tests were performed following the “Standard for Test Methods of Mechanical Properties of Ordinary Concrete” (GB/T50081-2016).

#### Crack healing test

For the crack healing evaluation of self-healing recycled concrete, prism specimens with dimensions of 100 × 100 × 400 mm were selected. Through-cracks were created by vertical cutting using a high-precision diamond saw, followed by fixing thin shims with thicknesses of 0.3, 0.5, and 0.7 mm at both ends of the cracks to control the initial crack width. Subsequently, the width of cracks at multiple locations was measured using a crack width microscope (1 DIV/0.02 mm), and specimens with actual crack widths deviating from the target values within ±0.02 mm were selected for subsequent healing experiments. To prevent additional damage during cutting, a low-speed, water-cooled method was used, and after cutting, high-pressure air was used to clean out debris from the cracks to ensure crack cleanliness and dimensional accuracy.

**Fig 5:**
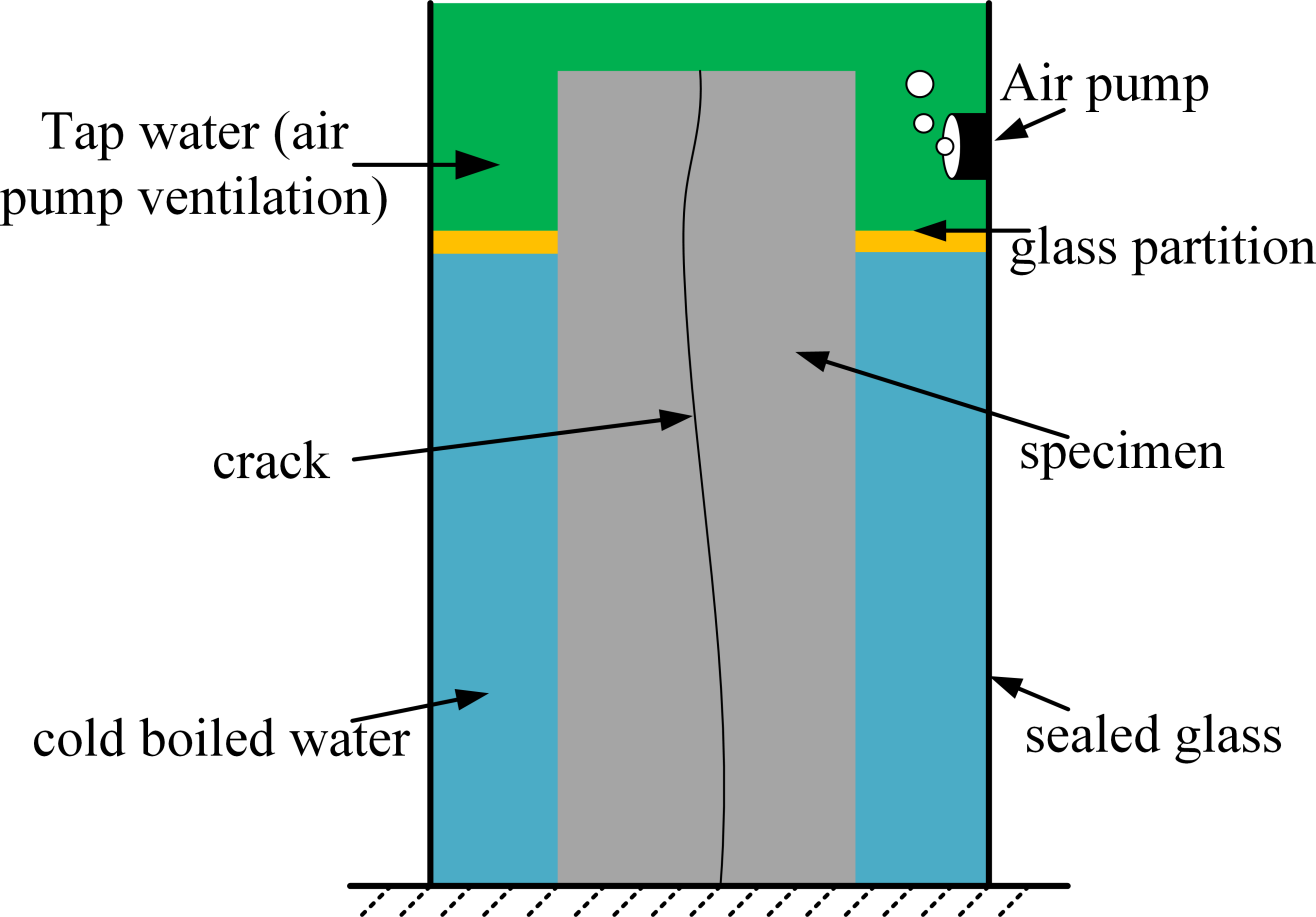
Crack healing setup.

The crack healing was conducted in a sealed glass container measuring 180 × 180 × 450 mm. A horizontal partition was installed 300 mm above the container bottom to separate the upper and lower sections (as shown in Fig. 5). The cracked specimen was placed vertically in the container. Boiled water cooled to room temperature was poured below the partition to reduce the oxygen content in the water, while tap water was injected above the partition, and a small air pump continuously injected air into the water to maintain a stable oxygen content. Different oxygen content regions were established within the healing system to simulate the heterogeneity of oxygen distribution in real concrete cracks and to investigate the effect of oxygen content on the crack healing performance of self-healing recycled concrete. The crack healing ratio was calculated using equation (1):


(1) $$\eta =\frac{W_{0}-W_{t}}{W_{0}}.$$

Where *W*_0_ represents the initial width of the crack, *W*_t_ denotes the residual width of the crack after t days of healing.

#### Compactness test

**Fig 6:**
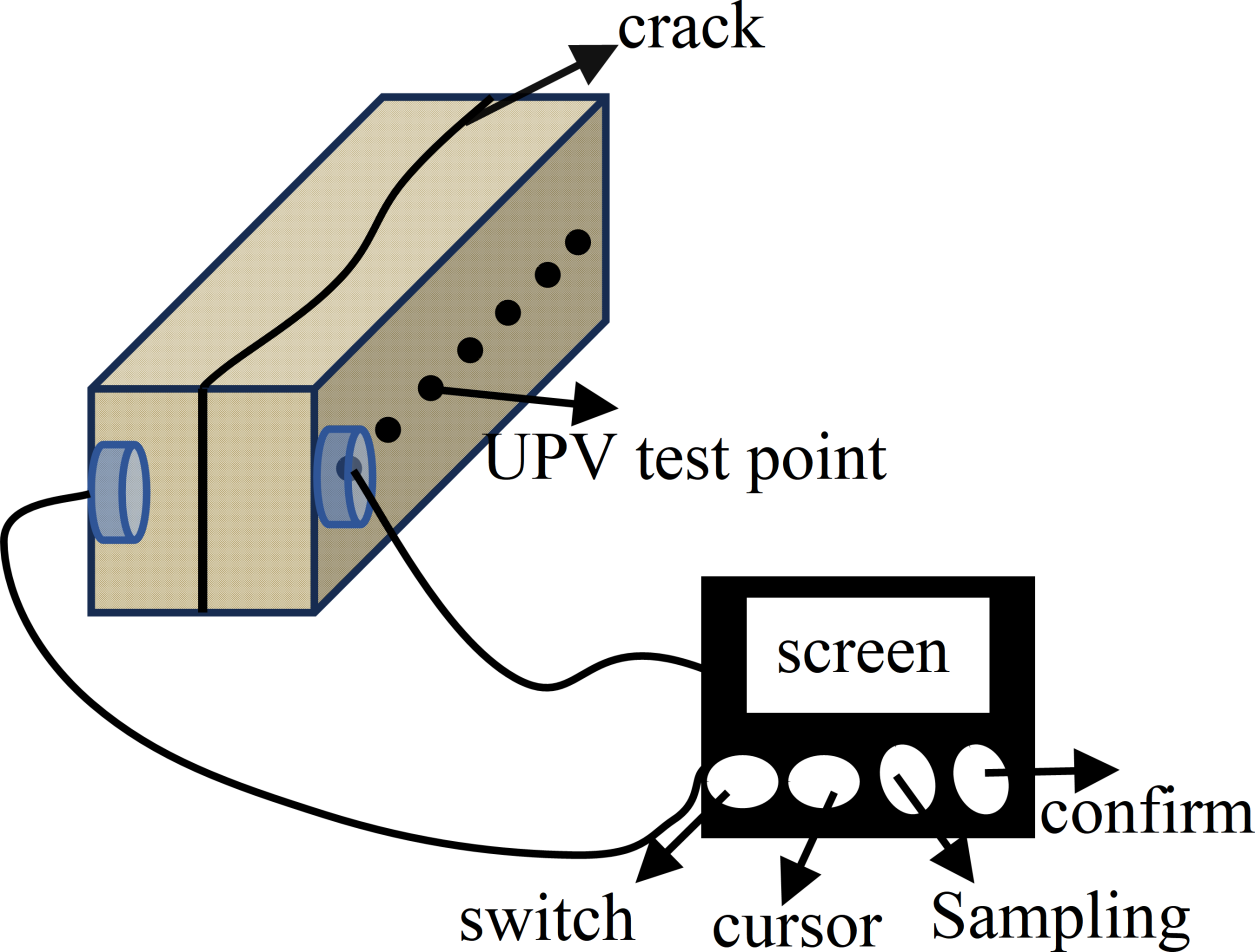
Schematic diagram of UPV.

The compactness of the specimen was tested using the UPV method (29). During testing, a pair of planar transducers (emitting and receiving) having a diameter of 50 mm were positioned on both sides of the crack. A non-test zone of 25 mm was reserved at each end of the specimen, and within the effective testing length, seven test points were arranged along the crack direction. The spacing between test points was determined based on the average detection length, and UPV tests were performed sequentially at these points, as shown in Fig. 6.

#### Microscopic performance test

After 28 days of healing, mineralized precipitates formed in the oxygen-rich, transition, and oxygen-deficient zones of R100-M5 specimens (5% dosage, 0.3 mm crack width) were collected for microstructural analysis. Microscopic experiments included SEM, EDS, XRD, FTIR, TG-DSC, and AFM. SEM analysis was performed using a Merlin Compact. Mineralized precipitates from three regions were prepared as ~10 mm block specimens and sputter-coated with gold prior to observation. Morphology was observed, and EDS was used to determine elemental composition. XRD analysis was conducted using a Rigaku Ultima IV diffractometer (Japan). The precipitates from three regions were ground, sieved through a 200-mesh screen, and tested under Cu Kα radiation (λ = 1.5406 Å) with a voltage of 40 kV, current of 40 mA, scanning range of 10°−80° (2θ), and scanning rate of 2°/min to identify crystalline phases. FTIR analysis was performed on powdered specimens mixed with KBr and pressed into transparent thin sheets. Spectra were collected in the range of 400–4,000 cm^−1^ to analyze functional groups. TG-DSC was conducted on 10–15 mg of dried powder samples placed in alumina crucibles under a nitrogen atmosphere with a constant flow of nitrogen gas. The heating rate was 10°C/min, and the temperature ranged from room temperature to 1,000°C to evaluate mass loss and quantify precipitates. AFM was performed on ethanol-cleaned and dried fragments in tapping mode using a micro-cantilever probe. Surface morphology was analyzed, and point adhesion force was measured to evaluate surface adhesion properties.

## RESULTS

### OD value measurement results

**Fig 7:**
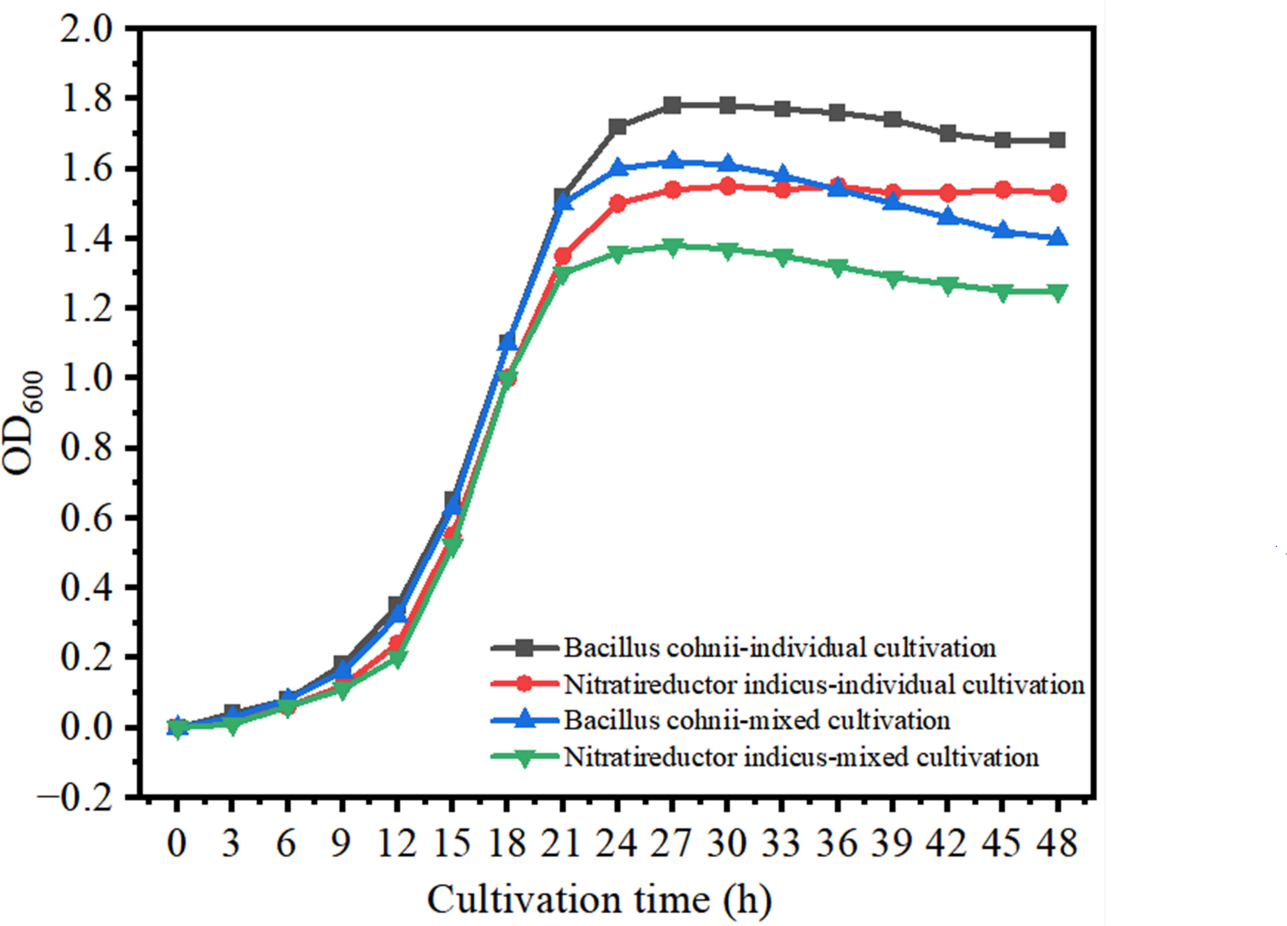
OD_600_ variation curves over time for separate and mixed cultivation.

As shown in Fig. 7, the time-dependent changes in OD_600_ of Bacillus cohnii and Nitratireductor indicus during individual and mixed cultivation.

It could be observed that under both individual and mixed cultivation conditions, the OD_600_ values over time differed for the two strains. In the mixed system, the OD_600_ values of both strains were lower than their respective values under individual cultivation throughout the cultivation period, indicating that mixed cultivation exerted a certain inhibitory effect on the growth of both strains.

### Mechanical performance

**Fig 8:**
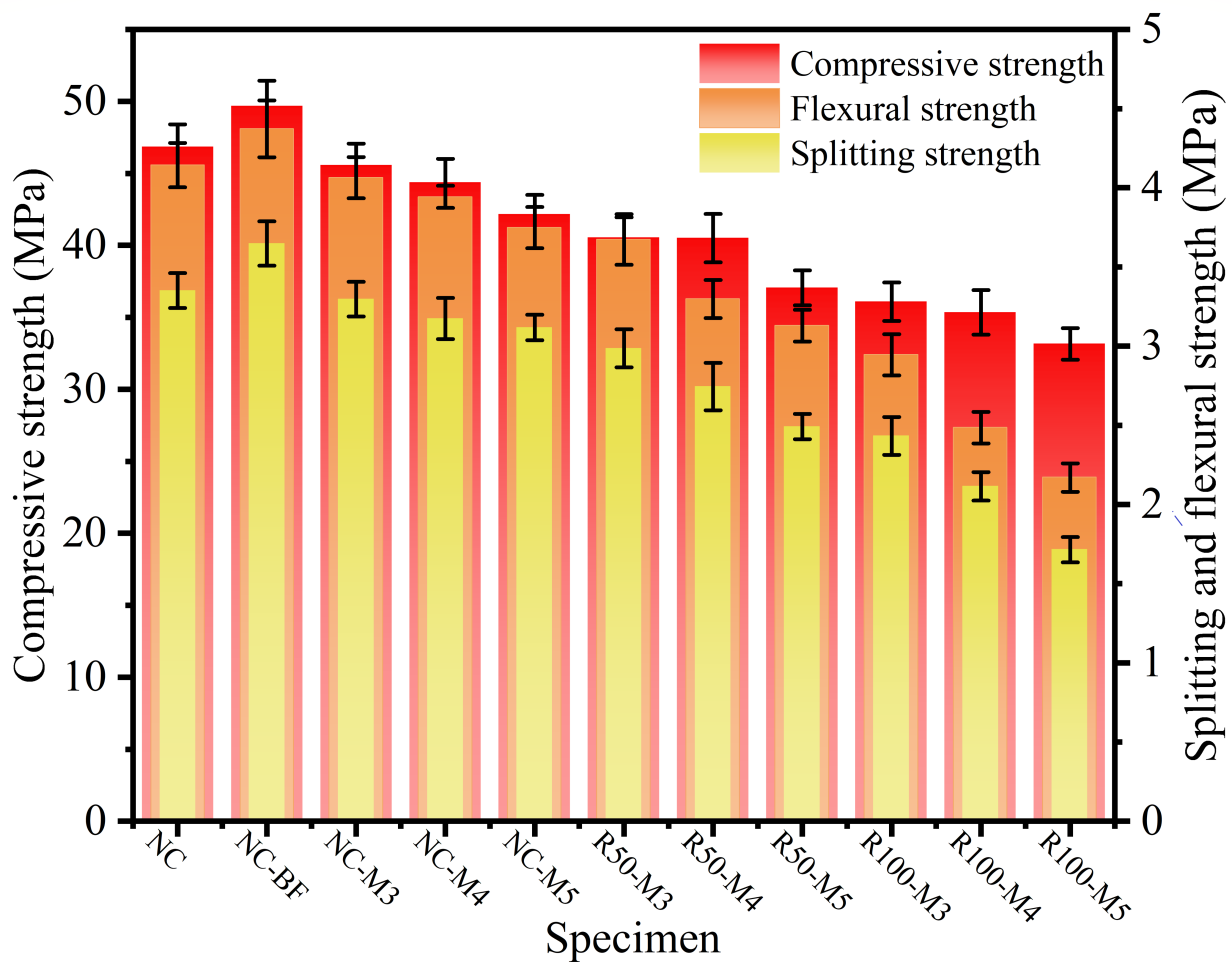
Mechanical strength of self-healing concrete.

Mechanical performance tests for self-healing recycled concrete mainly included cube compressive strength, splitting strength, and flexural strength tests. The results are shown in Fig. 8.

As shown in Fig. 8, when the repair agent dosage was 3%, the compressive strength of concrete with recycled aggregate replacement ratios of 0%, 50%, and 100% decreased by 8.3%, 18.4%, and 27.4%, respectively, compared with the NC-BF group with a dosage of 0%. At a 4% dosage, the reductions were 10.7%, 18.5%, and 28.9%, while at a 5% dosage, the decreases reached 15.2%, 25.5%, and 33.3%, respectively.

For the splitting and flexural strengths, when the dosage of the repair agent was 3%, the splitting strength of the concrete decreased by 9.6%, 18.1%, and 33.3% compared with the NC-BF group. At a dosage of 4%, it decreased by 13.0%, 24.7%, and 42.0%, and at a dosage of 5%, it decreased by 14.5%, 31.7%, and 52.9%. The flexural strength showed a similar trend. When the repair agent dosage was 3%, the concrete’s flexural strength decreased by 7.0%, 16.0%, and 32.6% compared with the NC-BF group. At a dosage of 4%, the flexural strength decreased by 9.8%, 24.6%, and 43.2%, and at a dosage of 5%, it decreased by 14.3%, 28.4%, and 50.4%. Compared to compressive strength, splitting and flexural strengths showed greater decline with increasing repair agent dosage and recycled aggregate replacement ratio.

**Fig 9:**
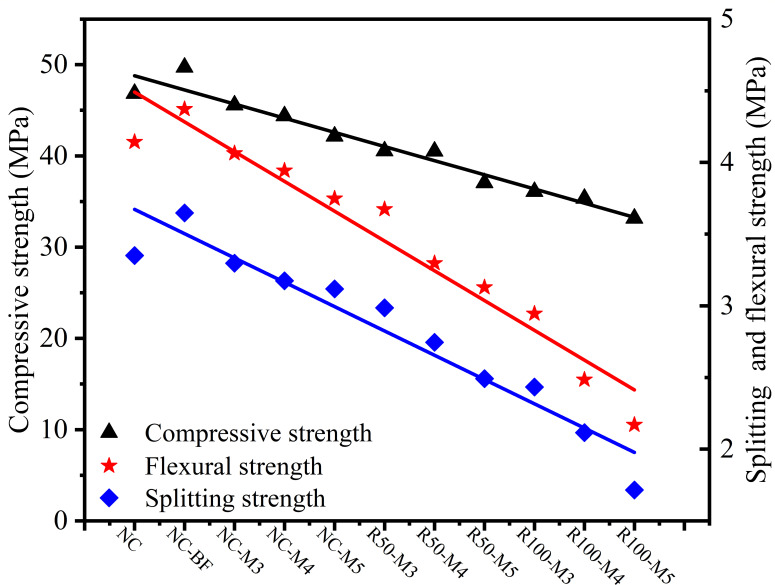
Mechanical performance fitted curve.

The relationships between repair agent dosage, replacement ratio, and mechanical properties were fitted using Origin software, and the fitting results are presented in Fig. 9.

It could be concluded that the fitted equations for compressive strength (*f*_c_), flexural strength (*f_f_*), and splitting strength (*f*_s_) were given by equations(equation 2), (equation 3), and (equation 4), respectively:


(2) $$f_{c}=48.61-1.20\;x_{a}-0.09\;x_{t}\;(R^{2}=0.9705),$$

(3) $$f_{f}=4.37-0.12\;x_{a}-0.01\;x_{t}\;(R^{2}=0.9374),$$

(4) $$f_{s}=3.60-0.10\;x_{a}-0.01\;x_{r}\;(R^{2}=0.9227).$$

Where x_a_ was the dosage of the repair agent, and x_r_ was the replacement ratio of recycled aggregate.

The correlation coefficients of the three fitted curves all exceeded 0.90, indicating that the formulas were reasonable. This also indicated that the repair agent dosage and the replacement ratio exhibited good linear correlation with the mechanical properties of self-healing recycled concrete.

### Healing effectiveness

**Fig 10:**
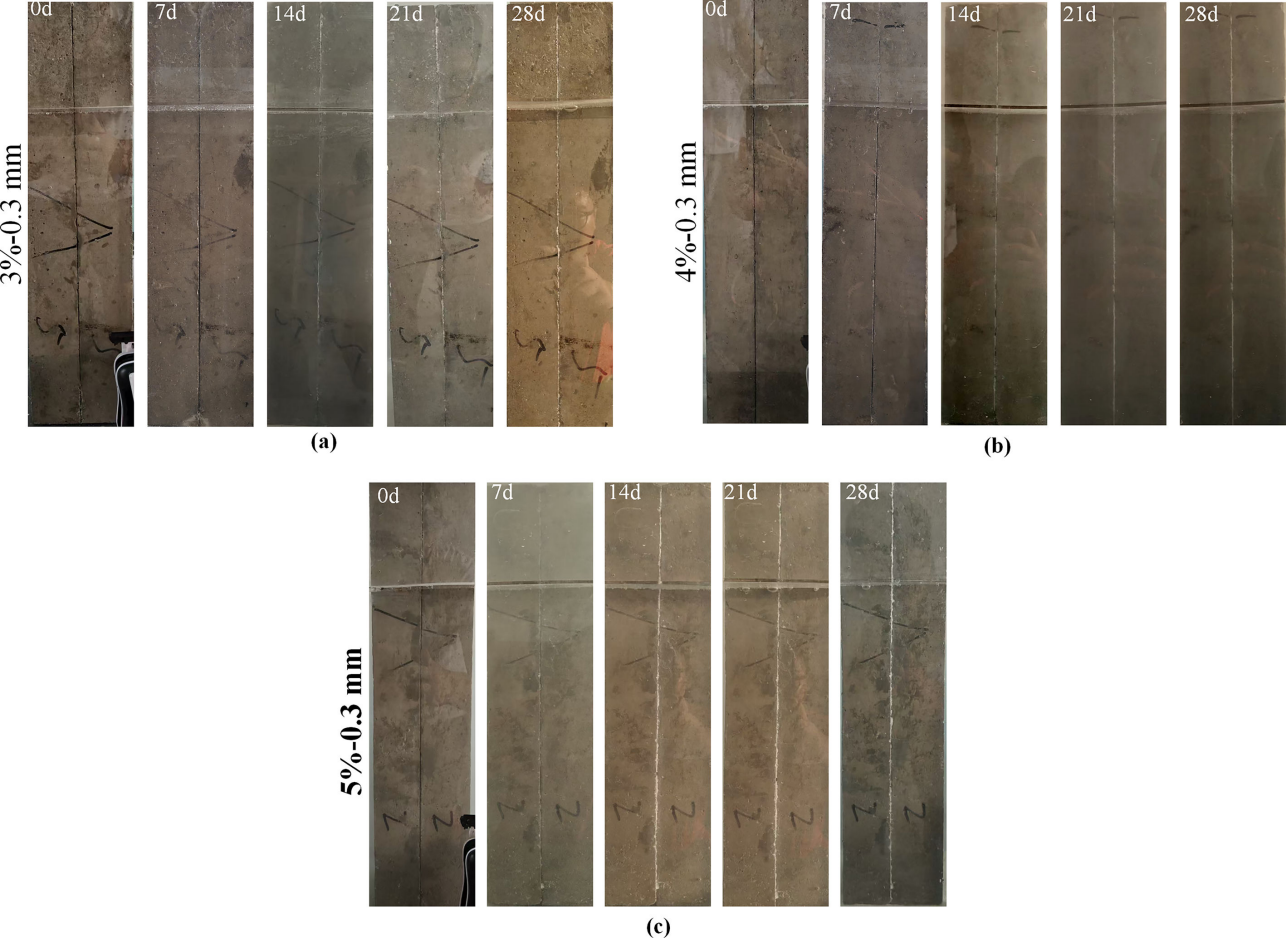
Repair effect images for self-healing recycled concrete groups. (**a**) R100-M3, (**b**) R100-M4, (**c**) R100-M5.

**Fig 11:**
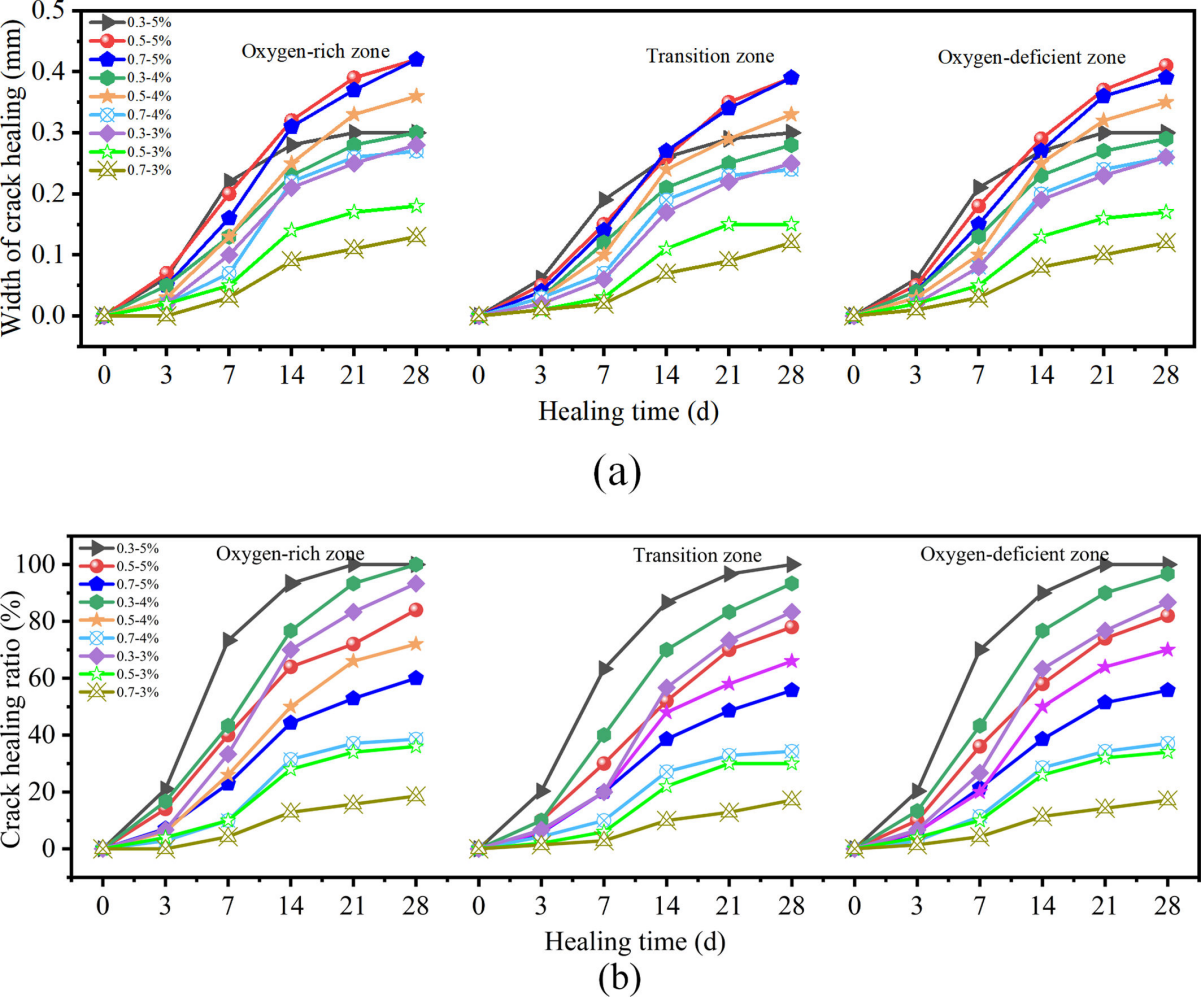
Variations in crack healing width (**a**) and healing ratio (**b**).

The carrier, serving as the habitat for microorganisms, significantly influences the healing effectiveness of cracks in concrete. The repair effects of different repair agent dosages when the crack width is 0.3 mm are shown in Fig. 10. The repaired crack widths and repair ratios under different crack widths and repair agent dosages are presented in Fig. 11.

Figure 10 illustrated that, as the healing process progressed, the concrete cracks gradually became filled with white precipitates. In the early stage of healing, the substances filling the cracks were relatively loose and not compact. As the curing age increased, the amount of microbial mineral deposition gradually increased, the crystals gradually became more densely packed, and there was a tendency to expand outward and protrude, resulting in a better crack healing performance.

As shown in Fig. 11, the crack self-healing performance gradually improved with decreasing crack width and increasing repair agent dosage. From day 0 to 3, the increase in healing ratio was relatively slow, with all healing ratios remaining below 25.00%. At this stage, the (0.3 mm, 5%) group exhibited the best performance, with a healed crack width of 0.063 mm and a healing ratio of 21.00%, whereas the (0.7 mm, 3%) group showed the poorest performance, with a healing ratio of only 1.43%. During the period of 3–14 days, the self-healing ratio increased most rapidly. During the 14–28 day period, the growth rate of the crack healing ratio gradually slowed compared to the 3–14 day period. After 28 days, there was essentially no significant change in the self-healing ratio.

After 28 days of healing, under different repair agent dosages (3%, 4%, and 5%), the healing ratios for 0.3 mm cracks in the oxygen-rich zone were 93.33%, 100.00%, and 100.00%, respectively; those in the transition zone were 83.30%, 93.30%, and 100.00%, while the corresponding values in the oxygen-deficient zone were 86.70%, 96.70%, and 100.00%. For 0.3 mm microcracks, high healing efficiency was achieved in all three zones, and as the repair agent dosage increased from 3% to 5%, the healing ratios approached or reached 100%, indicating that nearly complete healing of microcracks could be realized with sufficient repair agent. When the crack width increased to 0.5 mm, the healing ratios in the oxygen-rich zone at repair agent dosages of 3%, 4%, and 5% were 36.00%, 72.00%, and 84.00%, respectively; those in the transition zone were 30.00%, 66.00%, and 78.00%, while the corresponding values in the oxygen-deficient zone were 34.00%, 70.00%, and 82.00%. With increasing crack width, the healing ratios in all regions decreased significantly, but they still showed an upward trend with increasing repair agent dosage. Under cracks of 0.7 mm and the aforementioned dosage levels, the healing ratios in the oxygen-rich zone were 18.57%, 38.57%, and 60.00%, respectively; those in the transition zone were 17.14%, 34.29%, and 55.71%, while the corresponding values in the oxygen-deficient zone were 17.14%, 37.10%, and 55.71%. The healing capacity was further diminished under these conditions. Overall, the healing performance significantly decreased with increasing crack width, whereas increasing the dosage of the microbial repair agent could partially compensate for the adverse effect caused by larger crack widths.

**Fig 12:**
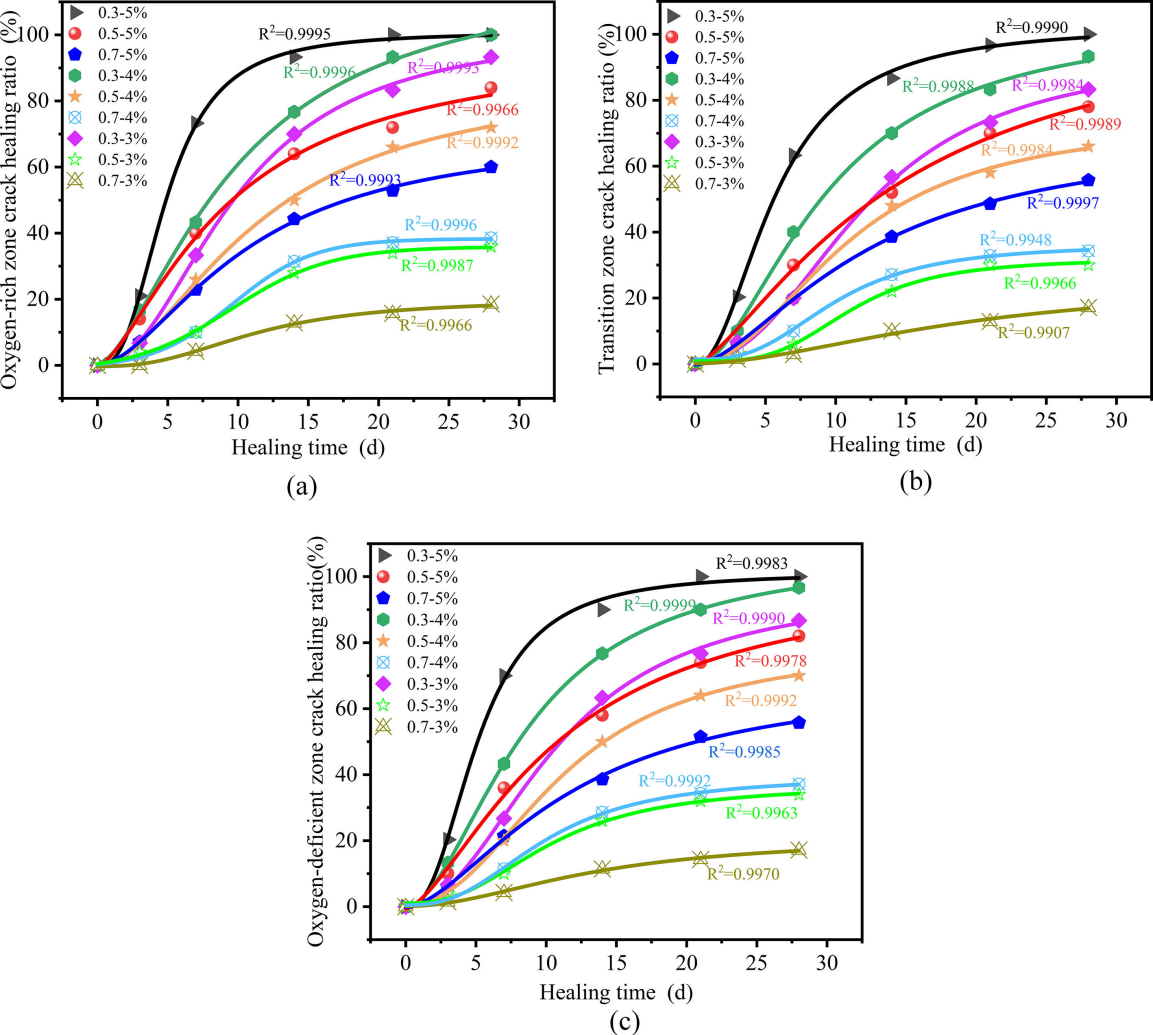
Fitted curves of healing ratio. (**a**) Oxygen-rich zone, (**b**) transition zone, (**c**) oxygen-deficient zone.

The variation of crack-healing ratio with time was fitted using the Logistic function in Origin software (as shown in Fig. 12). The correlation coefficients of the fitted curves for all three regions exceeded 0.99, indicating that the established model had high reliability. Moreover, the fitting results demonstrated that under different oxygen content zones, various combinations of repair agent dosages and crack widths, the healing ratio of self-healing recycled concrete over time exhibited a typical Logistic growth pattern, exhibiting pronounced nonlinear behavior.

**Fig 13:**
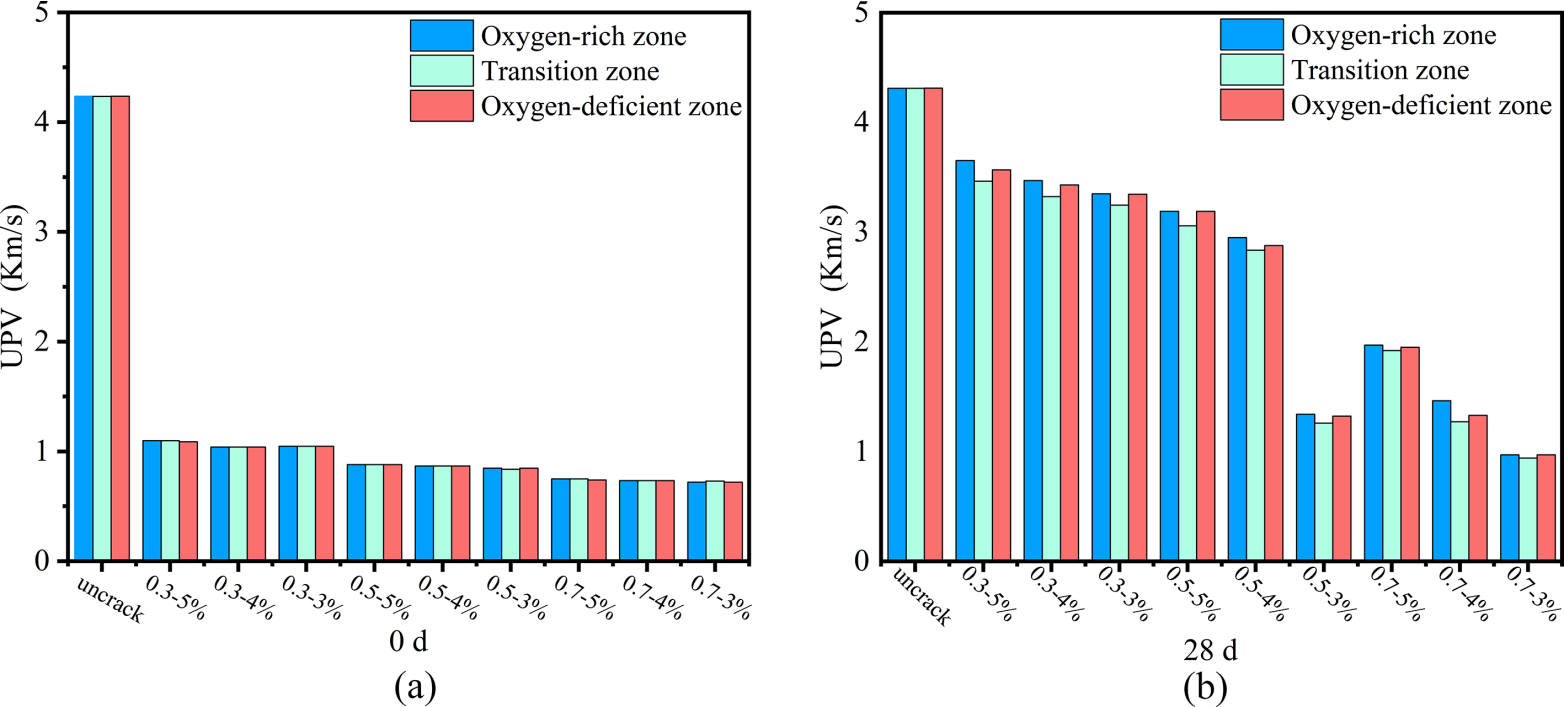
UPV under different healing times. (**a**) 0 day, (**b**) 28 days.

The compactness of the precipitates formed after crack healing was evaluated using ultrasonic testing and quantified in terms of UPV. Figure 13 presents the UPV results for specimens with different crack widths under various repair agent dosages at different healing durations.

As shown in Fig. 13, for the uncracked concrete specimens, the UPV increased slightly with increasing curing time, with an increment of only about 0.073 km/s, indicating a very limited improvement. For the cracked specimens, the crack surfaces in the self-healing recycled concrete were partially repaired, with white precipitates filling the cracks; however, due to the differences in repair agent dosage and crack width, varying degrees of healing were achieved, thereby leading to different UPV. According to the results shown in the figure, the concrete specimen with a crack width of 0.3 mm and a repair agent dosage of 5% exhibited the maximum increase in UPV, with increases of 2.556, 2.368, and 2.481 km/s in the oxygen-rich zone, transition zone, and oxygen-deficient zone, respectively. Compared with the pre-healing state, the UPV increased by factors of approximately 2.33, 2.16, and 2.28. In contrast, the concrete specimen with a crack width of 0.7 mm and a repair agent dosage of 3% showed the smallest UPV improvement, with increases of only 34.7%, 28.8%, and 34.7%, respectively.

### Healing mechanism

**Fig 14:**
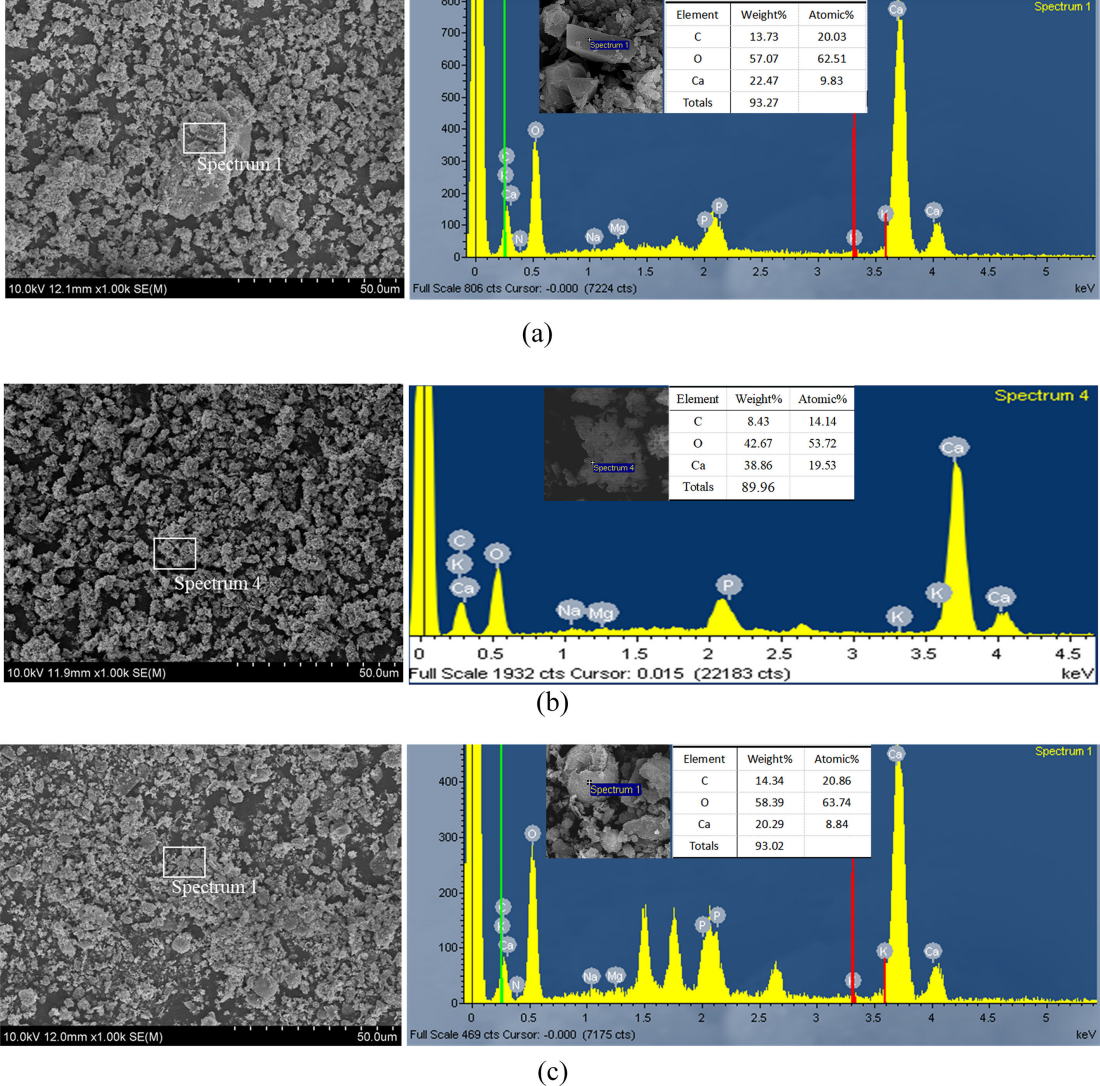
SEM image and EDS spectrum of mineralized precipitates at the crack. (**a**) Oxygen-rich zone, (**b**) transition zone, (**c**) oxygen-deficient zone.

The microscopic morphology and elemental composition of mineralized precipitates in different oxygen content zones were analyzed using SEM and EDS, as shown in Fig. 14. It was observed that the precipitates mainly exhibited cubic or irregular morphologies. Meanwhile, EDS analysis results indicated that the white precipitates in the three zones of the cracks were primarily composed of Ca, O, and C elements, confirming that the obtained mineralized precipitates were mainly composed of CaCO_3_. Minor amounts of other elements, such as N, Na, K, and Mg, were also detected in the precipitates.

**Fig 15:**
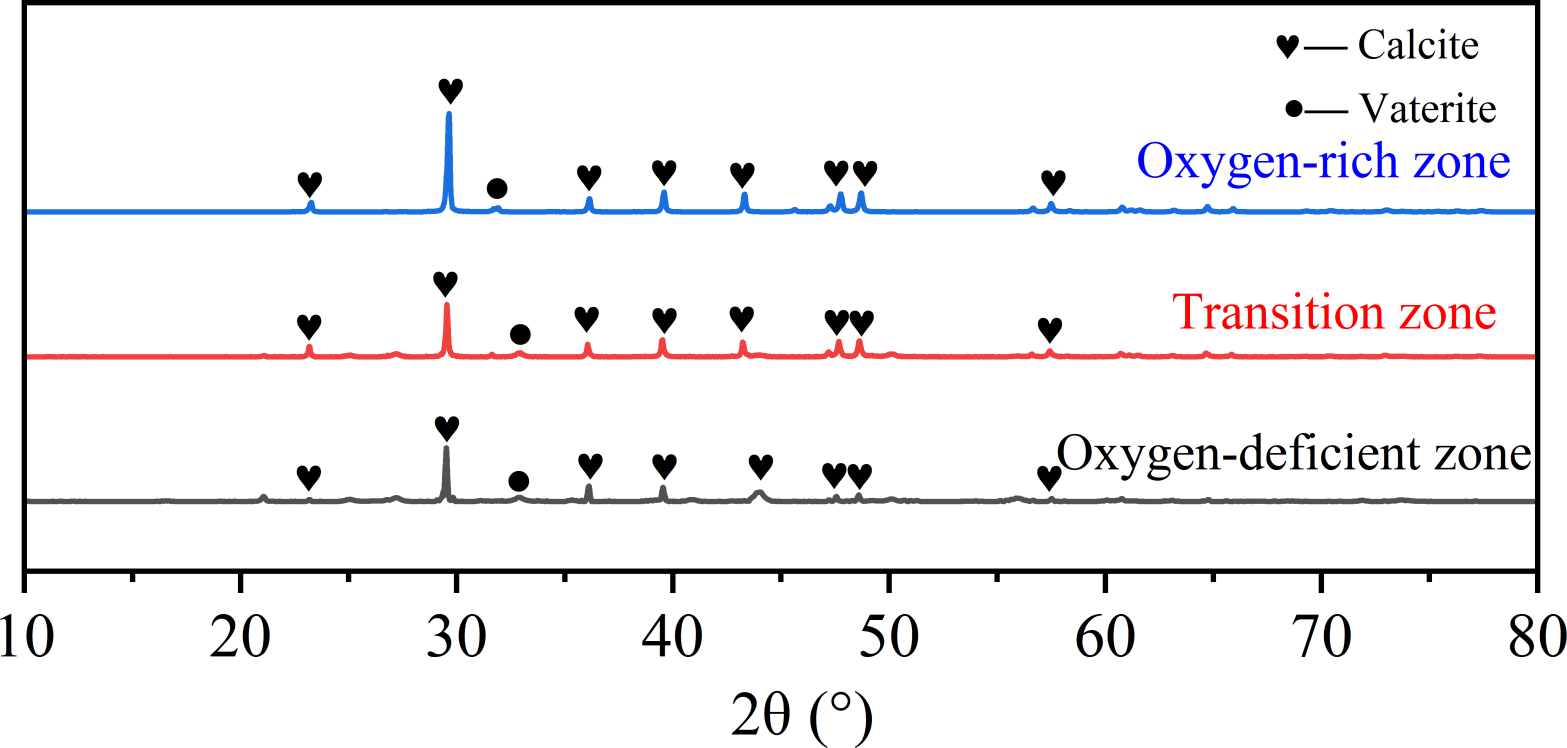
XRD pattern of mineral deposits at the crack.

To determine the crystal types of precipitates in different oxygen content zones, XRD analysis was performed on white precipitates from the oxygen-rich zone, transition zone, and oxygen-deficient zone, with results shown in Fig. 15. XRD analysis results indicated that the precipitates in all three zones were predominantly composed of calcite-type CaCO_3_, which was also consistent with the EDS analysis results. Furthermore, except for the diffraction peaks of CaCO_3_ polymorphs (calcite and trace vaterite), no diffraction peaks of other substances were found, indicating high purity of the mineralized healing precipitates. In addition, it was observed that the diffraction peaks in the oxygen-rich zone in Fig. 15 were higher and sharper compared to those in the other two zones.

**Fig 16:**
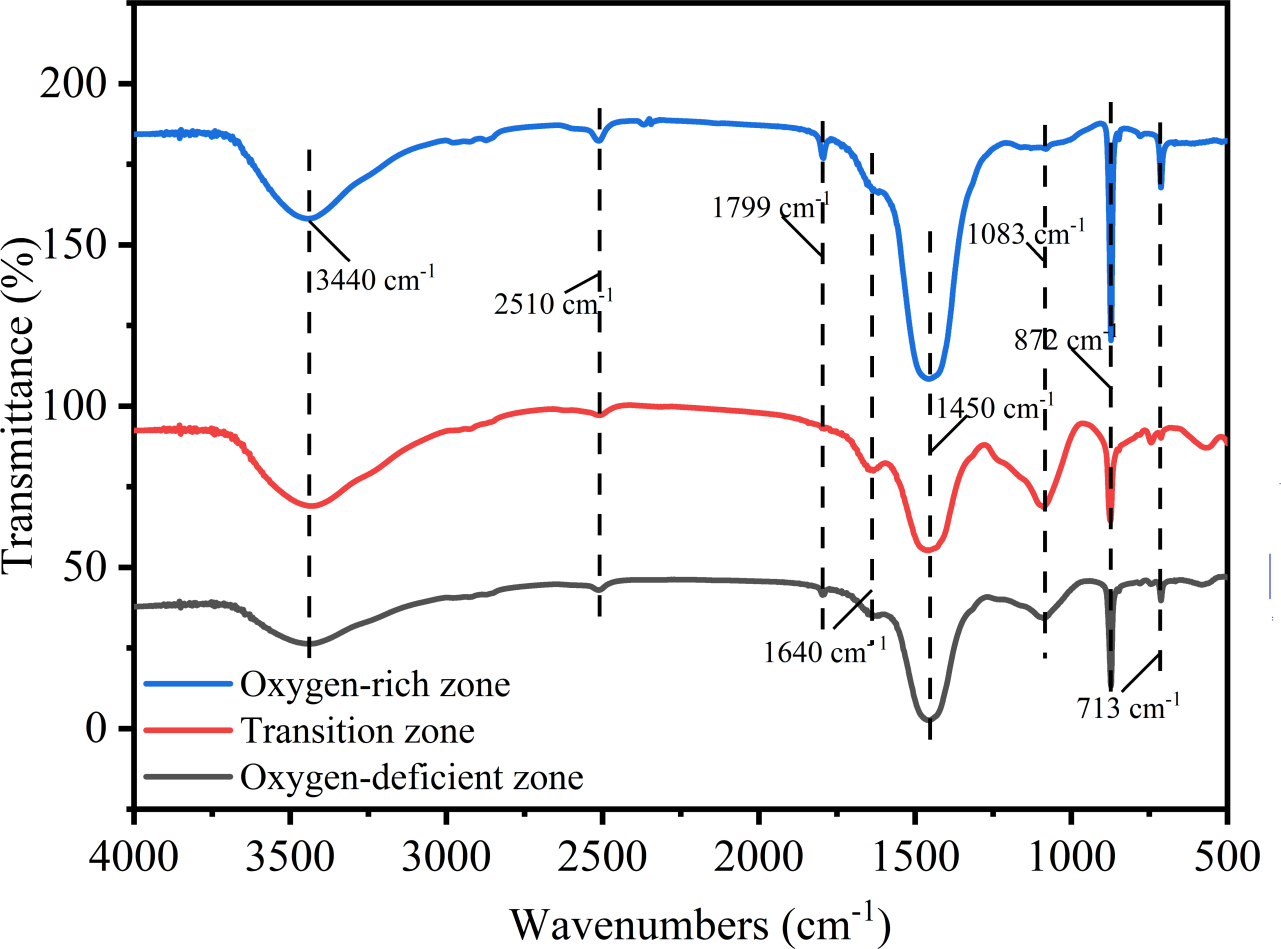
FTIR spectra of microbial mineralized precipitates from different zones.

The type of mineralized precipitates could also be analyzed by FTIR. FTIR spectra of microbial mineralized precipitates from different zones are shown in Fig. 16. As shown in the figure, the characteristic absorption peaks at 2,510 and 1,799 cm^−1^ were the combination frequency vibrations of CO_3_^2−^; the peak at 1,450 cm^−1^ was attributed to the asymmetric stretching of the C–O bond, and the characteristic absorption peaks at 872 and 713 cm^−1^ were mainly related to the bending vibrations of the C–O bond, indicating the presence of calcite-type CaCO_3_. In addition, other characteristic peaks were also observed at 3,440, 1,640, and 1,083 cm^−1^.

**Fig 17:**
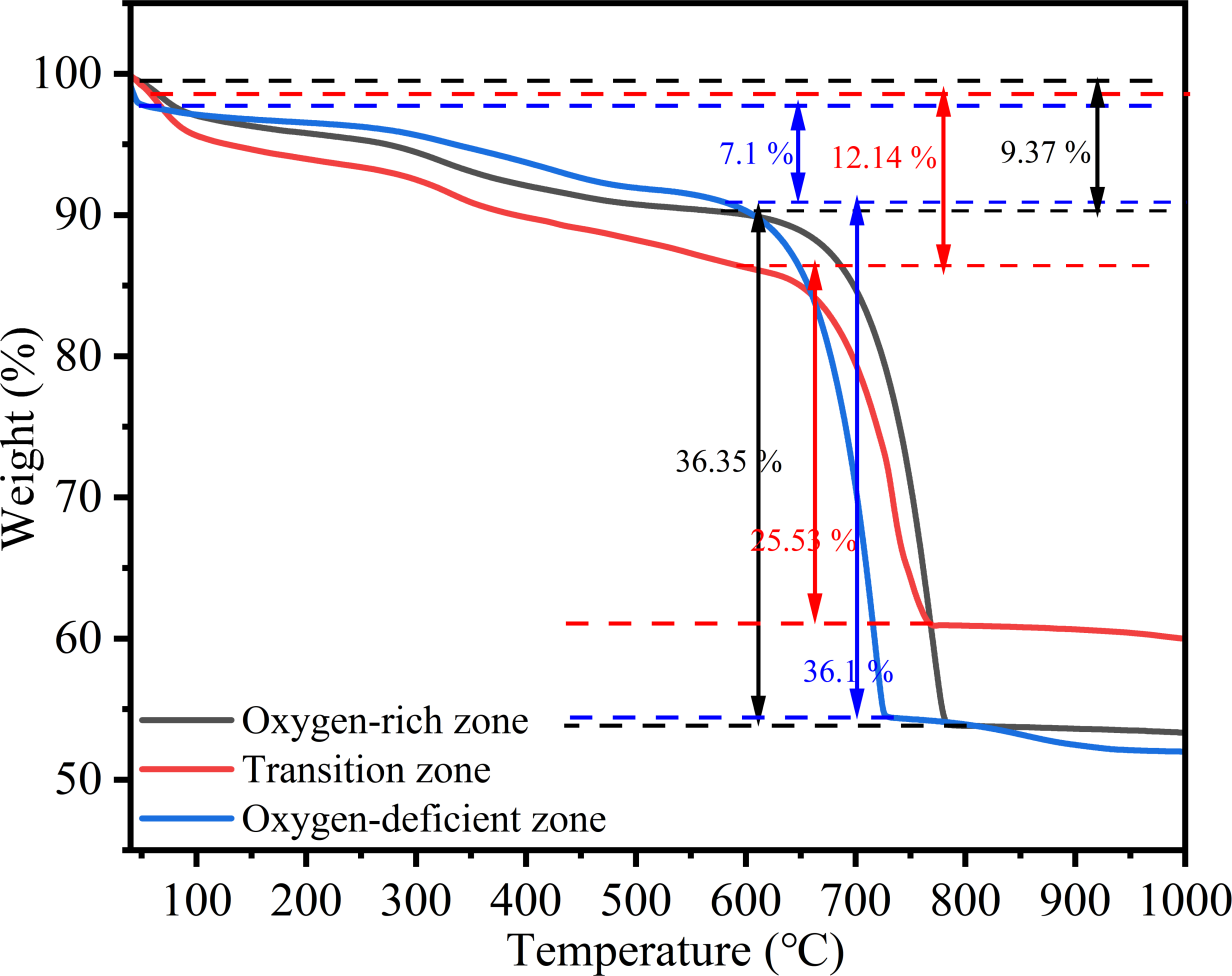
TG curves of microbial mineralized precipitates from different zones.

The composition of mineralized precipitates could also be analyzed by thermogravimetric analysis (TG), and the TG curves for zones with different oxygen content are shown in Fig. 17.

It was observed that the weight loss of mineralized precipitates exhibited similar variation patterns across different oxygen concentration zones, with all showing a two-stage weight loss process. The weight loss in the first stage occurred between 50°C and 600°C. This stage mainly involved the evaporation of free water and weakly bound water in mineralized precipitates, as well as the evaporation and decomposition of microbial proteins. The weight loss in the second stage occurred between 600°C and 800°C and was primarily attributed to CaCO_3_ decomposition. In this interval, the weight losses in the oxygen-rich zone, transition zone, and oxygen-deficient zone were 36.35%, 25.37%, and 36.10%, respectively. After 800°C, there was no significant weight loss in the oxygen-rich zone and the transition zone, but slight weight loss still occurred in the oxygen-deficient zone.

**Fig 18:**
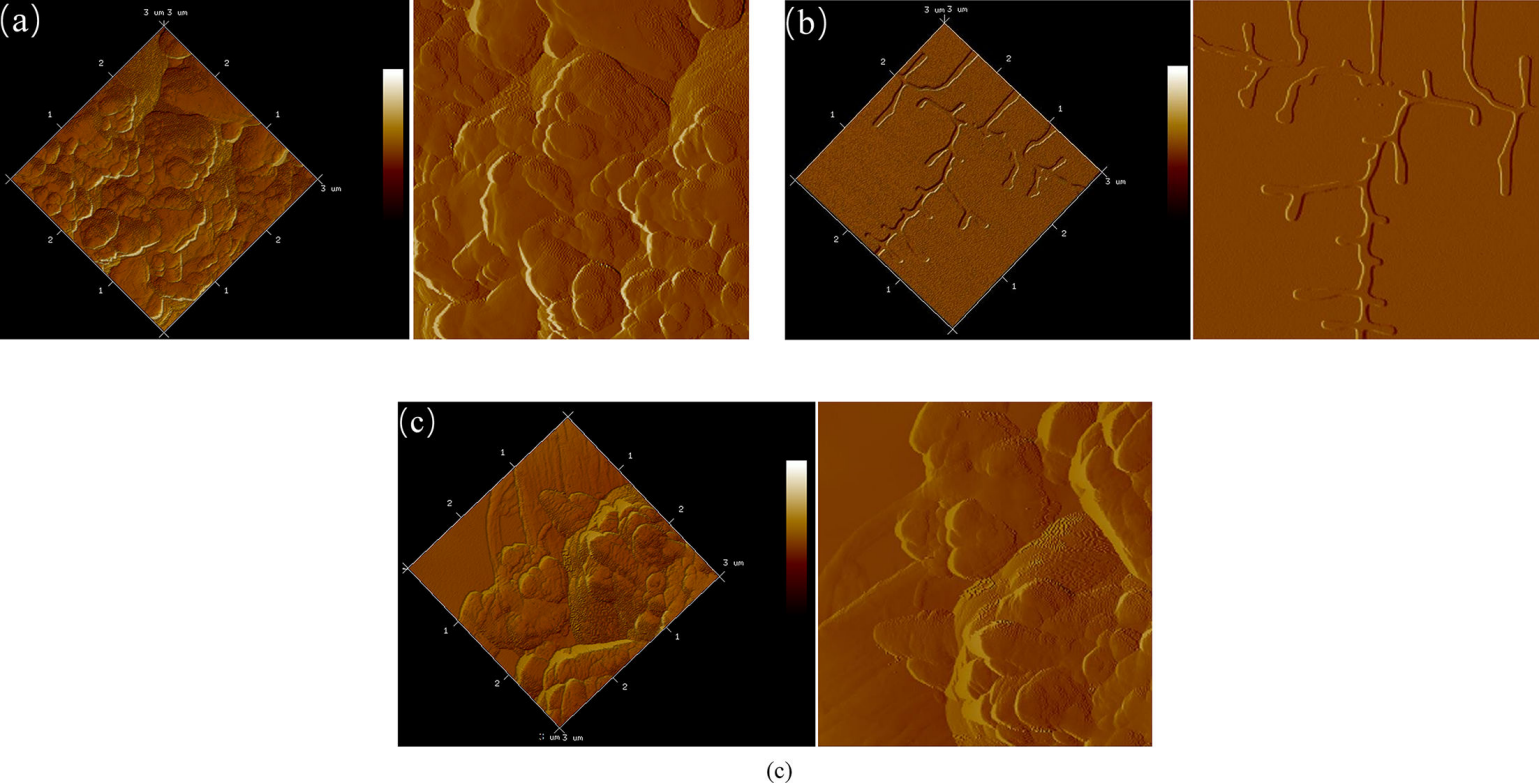
AFM testing of the morphology of mineralized precipitates. (**a**) Oxygen-rich zone, (**b**) transition zone, (**c**) oxygen-deficient zone.

**Fig 19:**
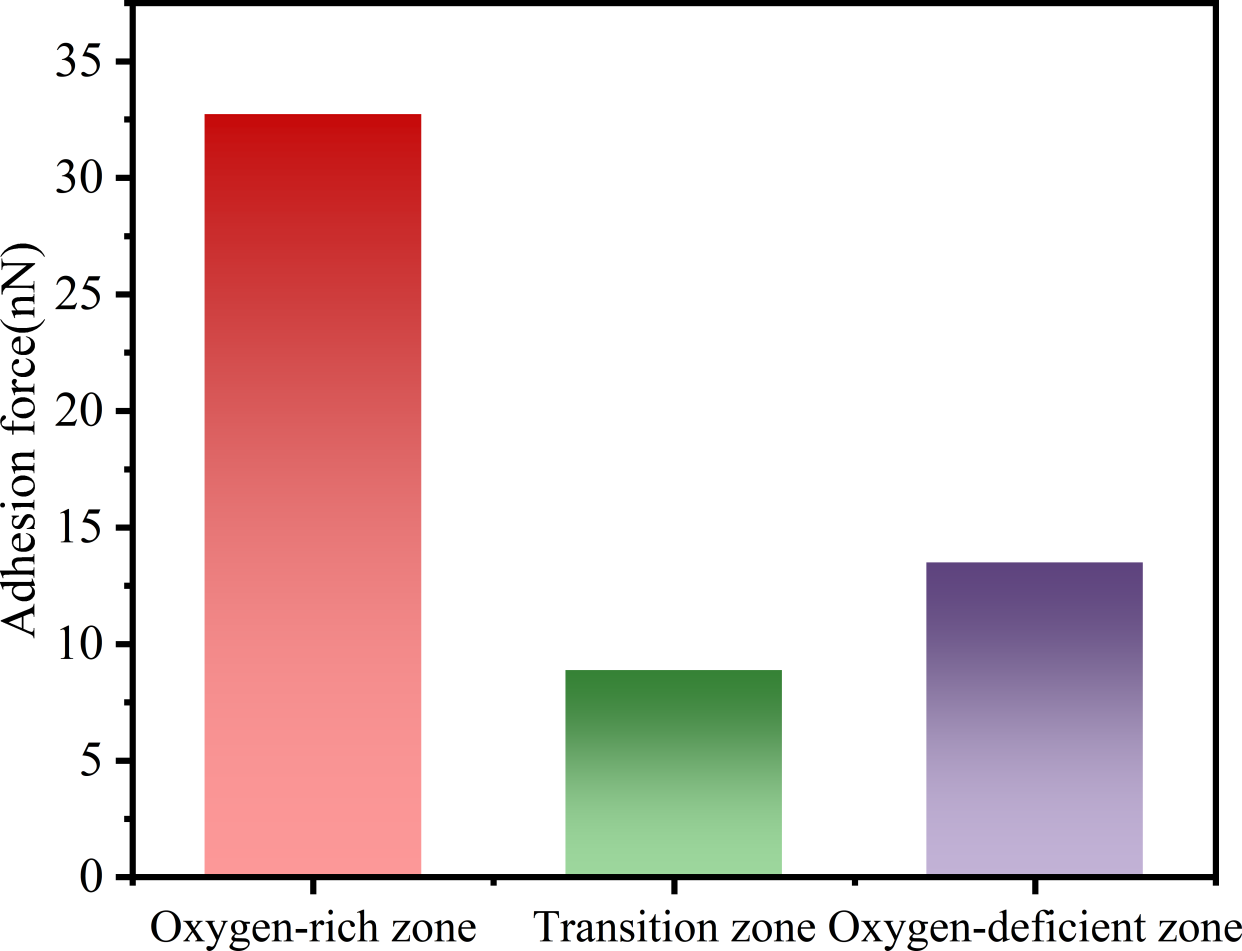
Adhesion force of microbially derived mineralized precipitates in different zones.

The adhesion force values in areas with different oxygen contents were measured using AFM. The microstructural features of the mineralized precipitates are illustrated in Fig. 18, and the adhesion forces of the mineralized precipitates are presented in Fig. 19 (the mean values of adhesion forces tested at five points on the morphology of the mineralized precipitates). As shown in Fig. 18, the morphology (3D images and relief images) of microbially induced mineralized precipitates in oxygen-rich and oxygen-deficient zones exhibited a certain degree of similarity. According to the analysis with NanoScope Analysis software, the morphology of the oxygen-rich zone mainly showed particles densely packed and evenly covering the surface, resulting in a relatively smooth surface; the morphology of the oxygen-deficient zone was mainly formed by the accumulation of granular structures, with the particles primarily being irregular aggregates and having a relatively rough surface. By comparison, the mineralized precipitates induced by microorganisms in the transition zone showed pronounced differences. The morphology in this area mainly appeared as flower-like or spiky spherical aggregates, and this complex and unique morphology often reflected special growth conditions.

Figure 19 revealed a pronounced disparity in the adhesion force of mineralized precipitates induced by microorganisms in different oxygen concentration zones. The adhesion forces of mineralized precipitates in the oxygen-rich and oxygen-deficient zones were 32.73 and 13.48 nN, respectively. By contrast, the adhesion force of microbially induced mineralized precipitates in the transition zone was further reduced compared to those in the oxygen-rich and oxygen-deficient zones, with an adhesion force of 8.86 nN in this region.

## DISCUSSION

### Influence of repair agent dosage and recycled aggregate replacement ratio on mechanical performance

This study demonstrated that the mechanical strength of concrete with added modified basalt fibers was higher than that without fibers. This was mainly because the surface roughness of the BF increased after treatment with a modified solution, which enhanced the bonding between the BF and the mortar matrix. In addition, the randomly distributed BF formed a three-dimensional spatial framework structure within the concrete, which inhibited the development of internal microcracks through a confinement effect. With the increase in the dosage of the repair agent and the replacement ratio of recycled aggregates, the mechanical strength of concrete showed a decreasing trend. This aligned with the results reported by Wang et al. (30, 31), Sheng et al. (32), Li et al. (33), and Jiang et al. (34). Wang et al. used recycled concrete aggregates and microcapsules as microbial carriers in self-healing concrete, while Li et al. and Jiang et al. used expanded perlite as the carrier and found that mechanical strength gradually decreased as the repair agent dosage increased. Similarly, Sheng et al. reported that excessive microbial carrier dosage also reduced compressive strength. Although their carrier materials and mix proportions differed from those used in this study, the strength variation trends were consistent with the present results. The main reason for this phenomenon was that during repair agent preparation, not only bacterial loading capacity but also timely crack response was considered. Due to the relatively low inherent strength of the repair agent, it easily became a weak point under axial stress. Additionally, recycled aggregates themselves had lower strength, higher water absorption, and weaker interfacial bonding (35, 36). This was mainly attributed to the residual adhered mortar on the surface of recycled aggregates and their high porosity, which increased internal voids, weakened the aggregate structure, and adversely affected both mechanical properties and the interfacial transition zone. As the replacement rate increased, internal defects in the concrete increased, further reducing its strength and thus causing a decrease in overall strength. Additionally, when increasing the dosage of microbial repair agent, the corresponding amounts of cement and sand in the same volume decreased, resulting in reduced mechanical strength of concrete.

Overall, the incorporation of the repair agent and variations in the replacement ratio exerted a weakening effect on the mechanical properties of concrete, and the extent of strength reduction intensified with increasing dosage. Although the addition of the repair agent was beneficial for crack healing, its negative impact on strength outweighed the reinforcing effect of BF, ultimately resulting in a reduction in strength. This trend was more pronounced than that observed for compressive strength.

### Healing effectiveness analysis

This study demonstrated that the crack healing ratio was relatively low during the first 3 days. This might be due to the fact that this stage belonged to the initial cracking phase of concrete, where bacterial spores immobilized in the carrier were in a dormant state requiring time for activation and metabolic restoration. Additionally, the alkaline environment within concrete inhibited microbial activities, only activating and enabling the function of surface-sensitive bacteria, resulting in low mineralization efficiency and reduced mineralized precipitation yield. During the period of 3–14 days, the self-healing ratio increased most rapidly, and the bacteria gradually adapted to the internal concrete environment, leading to enhanced metabolic activity and mineralization capability. Meanwhile, during this stage, the supply of moisture and calcium sources was sufficient, resulting in increased production of mineral precipitates. During the 14–28 day period, the growth rate of the crack healing ratio gradually slowed compared to the 3–14 day period. This might be due to the gradual filling of cracks by mineral precipitates, which narrowed the crack channels and limited the efficiency of moisture and calcium transport, thereby slowing down the microbial mineralization ratio. After 28 days, there was essentially no significant change, primarily because for 0.3 mm cracks, the precipitates had largely filled the crack channels, blocking the transport of moisture and calcium sources, thereby interrupting microbial mineralization reactions. For 0.5 and 0.7 mm wider cracks, precipitates covered the crack surfaces, limiting bacterial dissolution and further mineralization. Consequently, the self-healing rate remained nearly constant after 28 days.

The slight increase in UPV observed in the uncracked specimens was mainly attributed to the continued hydration of unhydrated cement particles. After 28 days of healing, the UPV of cracked concrete specimens in all three zones increased to varying extents. The observed UPV recovery was consistent with previous studies. For example, Arunachalam (37) reported a maximum UPV increase of 28% for 0.5–1.0 mm cracks healed by Bacillus tropicus. This was highly consistent with our 0.7 mm crack specimen (which showed a 34.7% improvement). However, our narrower 0.3 mm cracks achieved a much higher recovery, highlighting the significant impact of crack width. In addition, Kaur et al. (38) investigated the healing of 0.6 mm cracks using ultrasonic monitoring techniques and observed a significant increase in signal amplitude during the healing process. At the end of healing, the amplitude ratio approached that of uncracked specimens, further confirming the effectiveness of ultrasonic techniques in evaluating crack repair performance.

The results indicated that after microbial self-healing treatment, the UPV of the specimens was partially restored but did not reach the initial UPV value of the uncracked specimens. This was mainly because the microbial mineralized precipitates were predominantly CaCO_3_. Although CaCO_3_ precipitates could fill the cracks, differences in elastic modulus, acoustic impedance, and microstructural characteristics between CaCO_3_ and the concrete matrix, as well as the possible presence of unfilled micro-voids within the repaired crack zones, made it difficult to achieve complete densification. Consequently, the UPV remained lower than the initial value of the uncracked specimens. As shown in Fig. 13, all three zones exhibited higher UPV values with increasing repair agent dosage and decreasing crack width. This trend was consistent with the results observed for crack healing width and healing ratio. A higher UPV indicated greater compactness of the mineralized precipitates within the cracks, thereby reflecting a more effective self-healing performance.

### Microstructural characterization and mineralization mechanism analysis

The predominance of Ca, C, and O confirmed that the precipitates were mainly CaCO_3_, which was consistent with previous studies by Cheng et al. (39) and Oh et al. (40). Cheng et al. reported predominantly cubic and blocky calcite crystals, whereas Oh et al. observed irregular clustered precipitates, both consistent with the morphologies observed in this study. It was noteworthy that the atomic ratios of the elements did not strictly follow the theoretical chemical stoichiometric ratio of CaCO_3_ (1:1:3), and minor amounts of elements, such as N, Na, K, and Mg, were detected, which might be attributed to the characteristic features of microbial involvement in biomineralization processes. The presence of N elements and the non-stoichiometric characteristics of C and O proportions were primarily attributed to extracellular polymeric substances (EPS) and proteins secreted by microorganisms during growth and mineralization processes. These organic materials were either encapsulated within the crystal or adhered to its surface, contributing additional C, O, and characteristic N element signals. In addition, trace elements, such as Na, K, and Mg, mainly originated from the microbial culture medium and residues of modified solutions. Due to the extremely high solubility of Na_2_CO_3_ and K_2_CO_3_, it was difficult for them to crystallize stably in the moist crack environment. Consequently, Na and K elements mainly existed in ionic form adsorbed on the calcium carbonate surface or encapsulated within microbial organic matter (EPS), whereas Mg^2+^ predominantly occurred in trace adsorbed or lattice substitution forms.

**Fig 20:**
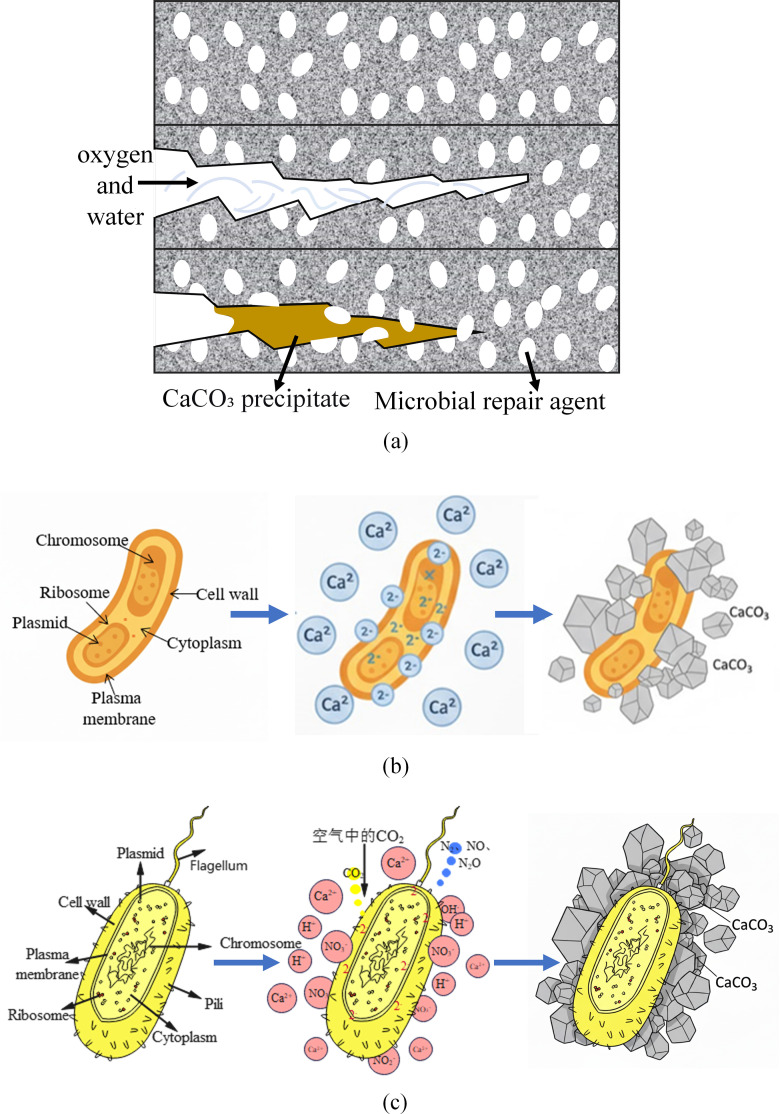
Schematic diagram of the crack healing mechanism in self-healing recycled concrete. (**a**) Crack healing diagram, (**b**) aerobic bacteria mineralization reaction, (**c**) denitrifying bacteria mineralization reaction.

The XRD results indicated that the mineralized precipitates were mainly composed of calcite-type CaCO_3_, which agreed well with the findings of Su et al. (41), who reported that bacterial mineralized precipitates in fiber-reinforced systems were predominantly calcite, with no other detectable crystalline phases. In their study, CaCO_3_ precipitated on polypropylene (PP) fibers via microbial activity and EPS-mediated nucleation, forming highly crystalline deposits. The diffraction peaks in the oxygen-rich zone in Fig. 15 were higher and sharper compared to those in the other two zones, indicating that the content of healing precipitates and their crystallinity were superior to those in the other two zones. The primary reason was that moisture and oxygen from the external environment entered the concrete through crack channels. The oxygen-rich zone, due to ample oxygen supply, activated the dormant microbes pre-embedded within the concrete, initiating a mineralization reaction between them and the substrate; the oxygen-deficient zone, due to the low oxygen content, hindered the participation of aerobic microorganisms in mineralization reactions, thereby activating anaerobic microorganisms to perform denitrification and generate mineralized precipitates. However, compared to aerobic bacteria, the mineralization reactions of anaerobic bacteria proceeded more slowly. The transition zone was essentially the same as the oxygen-rich and oxygen-deficient zones, but because it was in a transitional stage of oxygen concentration, the oxygen content was relatively low. Aerobic bacteria did not actively carry out mineralization reactions, and anaerobic bacteria also found it difficult to have favorable conditions for mineralization and deposition. The reaction mechanism is shown in Fig. 20.

The obtained FTIR results were consistent with previous studies by Xiang et al. (42) and Zhang et al. (43). In these studies, vibration peaks related to CaCO_3_ (e.g., ~1,430, ~870, and ~710 cm^−1^) were observed, along with other peaks attributed to microbial metabolites (e.g., ~3,457 and ~1,632 cm^−1^), indicating that calcite exhibited similar FTIR features in microbial-induced mineralization systems. Compared with these studies, despite the use of different microorganisms, the FTIR characteristics of the mineralized precipitates remained highly consistent, suggesting that calcite was the dominant mineral phase. The XRD analysis provided further support for this finding (Fig. 15), confirming that the obtained mineralized precipitates were calcite-type CaCO_3_. As shown in Fig. 16, there were significant differences in the peaks at 1,799 cm^−1^ among the three zones. Both the oxygen-rich and oxygen-deficient zones exhibited distinct peaks, whereas the transition zone showed relatively flat profiles. In this zone, the mineralization efficiency of aerobic bacteria was reduced due to the decreased oxygen concentration, and anaerobic bacteria were affected by the interference of oxygen. The unique environmental conditions led to low crystallinity of mineralized precipitates, preventing the formation of the combination frequency vibration peaks characteristic of high crystallinity. Since the band at 1,799 cm^−1^ was particularly sensitive to crystallinity, this phenomenon could be clearly observed. This was also consistent with the XRD analysis. Other characteristic peaks were observed, such as the 3,440 cm^−1^ peak attributed to O–H stretching vibration, the symmetric stretching peak of microbial-secreted proteins at 1,640 cm^−1^, and the asymmetric stretching characteristic absorption peak of phosphate groups in microbial-secreted proteins at 1,083 cm^−1^. These results suggested the existence of microbial traces in CaCO_3_.

The first stage weight loss (50°C–600°C) was mainly attributed to the evaporation of free and weakly bound water, as well as the decomposition of microbial organic substances, such as proteins and polysaccharides. The evaporation of free water usually occurred between 50°C and 150°C, while the evaporation of loosely bound water mostly occurred between 150°C and 300°C. Above 300°C, organic substances began to decompose, which was consistent with the findings of Sönmez Tugluca et al. (44), who reported a similar decomposition temperature range. The N element detected by EDS suggested the presence of microbial organic substances, which were consistent with the characteristic peaks at 3,440, 1,640, and 1,083 cm^−1^ in the FTIR spectrum. Meanwhile, the FTIR spectra and the first-stage mass loss from TG analysis indicated that precipitates in the transition zone contained higher proportions of free water, weakly bound water, and organic residues compared to those in the oxygen-rich and oxygen-deficient zones. The second stage weight loss (600°C–800°C) corresponded to the decomposition of calcite-type CaCO_3_, which was consistent with the XRD and FTIR results confirming calcite as the dominant crystalline phase, as also reported by Liu et al. (45). Liu et al. employed Bacillus subtilis and reported that the mineralized precipitates were mainly composed of calcite-type CaCO_3_, with a weight loss of approximately 34.11% in the temperature range of 600°C–810°C due to CaCO_3_ thermal decomposition. A similar weight loss was observed within the same temperature range in the present study, further confirming that the main crystalline phase of the mineralized precipitates is calcite-type CaCO_3_. After 800°C, there was no significant weight loss in the oxygen-rich zone and the transition zone, but slight weight loss still occurred in the oxygen-deficient zone. This phenomenon primarily arose from the possible presence of small amounts of nitrogen-containing organic residues encapsulated by calcium carbonate, as well as thermally stable organic-inorganic composites in the oxygen-deficient zone. These components underwent further decomposition above 800°C, causing a slight additional weight loss. The CaCO_3_ in the oxygen-rich zone was denser, exhibited higher crystallinity, and contained relatively less organic matter. In the transition zone, the CaCO_3_ content was relatively low, and the organic matter had already been released during the low-temperature stage, so there was no significant additional weight loss after 800°C. Based on the combined analysis of FTIR and EDS spectra, as well as TG curves, it could be concluded that microorganisms participated in the formation of CaCO_3_ and were crucial to the nucleation and growth stages.

The AFM results showed that the mineralized precipitates exhibited distinct morphological differences among the three zones. These observations were in agreement with the SEM results presented in Fig. 14, further confirming the morphological differences of mineralized precipitates formed in regions with different oxygen availability. The differences in adhesion force were primarily attributed to variations in microstructural morphology, crystallinity, and compactness among mineralized precipitates across different zones. The smooth and dense surface of the oxygen-rich zone provided a larger effective contact area, thereby maximizing the van der Waals forces at the probe-sample surface, and thus resulting in the maximum adhesion force; in contrast, the mineralized precipitates in the oxygen-deficient zone showed looser interparticle bonding and a rougher surface morphology. The irregular surface diminished the effective contact area at the probe-sample interface, leading to a lower adhesion force. For the transition zone, the mineralized precipitates were characterized by extremely low crystallinity and compactness, as evidenced by the disappearance of the characteristic FTIR peak at 1,799 cm^−1^. This loose structure significantly reduced the effective contact points of the probe, thereby leading to the lowest adhesion force.

### Conclusion

This study mainly investigated the mechanical properties and crack self-healing performance of microbial self-healing recycled concrete, and the main conclusions were as follows:

1. The incorporation of basalt fiber effectively enhanced the strength of concrete. However, with increasing repair agent dosage and recycled aggregate replacement ratio, the compressive, splitting, and flexural strengths of concrete exhibited decreasing trends. Compared to compressive strength, the reductions in splitting and flexural strengths were more pronounced.
2. The crack-healing performance improved with increasing repair agent dosage. For narrow cracks (0.3 mm), all three zones exhibited high healing efficiency, and as the repair agent dosage increased from 3% to 5%, the healing ratios approached or reached 100%, indicating that microcracks could be almost completely healed under sufficient repair agent dosage. With increasing crack width, the healing ratios in all zones decreased significantly, but they still showed an increasing trend with increasing repair agent dosage.
3. After 28 days of healing, the UPV of specimens with all crack widths and repair agent dosages showed increases, indicating that cracks were partially filled by microbially induced CaCO_3_ precipitates and that internal compactness was improved. However, the UPV after healing remained lower than the initial value of uncracked specimens. For the same crack width, the increase in UPV became more pronounced with increasing repair agent dosage, which was consistent with the observed trends in crack healing ratios.
4. The mineralized precipitates formed in the oxygen-rich zone, transition zone, and oxygen-deficient zone were all identified as calcite-type CaCO_3_. Although the mineral phases were consistent across the three zones, there were still significant differences in crystallinity, microscopic morphology, and content. The oxygen-rich zone exhibited the highest crystallinity, content, and adhesion force, followed by the oxygen-deficient zone, while the transition zone showed the poorest performance. This further demonstrated that the effectiveness of microbial mineralization performance was optimal in the oxygen-rich zone, followed by the oxygen-deficient zone, with the transition zone being the least effective.

## Data Availability

Data will be made available on request.
